# Gold and Cobalt Oxide Nanoparticles Modified Poly-Propylene Poly-Ethylene Glycol Membranes in Poly (ε-Caprolactone) Conduits Enhance Nerve Regeneration in the Sciatic Nerve of Healthy Rats

**DOI:** 10.3390/ijms22137146

**Published:** 2021-07-01

**Authors:** Derya Burcu Hazer Rosberg, Baki Hazer, Lena Stenberg, Lars B. Dahlin

**Affiliations:** 1 Department of Hand Surgery, Skåne University Hospital, 205 02 Malmö, Sweden; lena.stenberg@med.lu.se (L.S.); lars.dahlin@med.lu.se (L.B.D.); 2 Department of Translational Medicine—Hand Surgery, Lund University, 205 02 Malmö, Sweden; 3 Department of Neurosurgery, Mugla Sitki Kocman University, Mugla 48100, Turkey; 4 Department of Aircraft Airflame Engine Maintenance, Kapadokya University, Ürgüp 50420, Turkey; bhazer2@yahoo.com; 5 Department of Chemistry, Zonguldak Bülent Ecevit University, Zonguldak 67100, Turkey; 6 Department of Biomedical and Clinical Sciences, Linköping University, 581 83 Linköping, Sweden

**Keywords:** axonal regeneration, nerve conduits, poly-caprolactone, poly-ethylene glycol, gold nanoparticle, cobalt oxide nanoparticle, heat-shock protein 27

## Abstract

Reconstruction of nerve defects is a clinical challenge. Autologous nerve grafts as the gold standard treatment may result in an incomplete restoration of extremity function. Biosynthetic nerve conduits are studied widely, but still have limitations. Here, we reconstructed a 10 mm sciatic nerve defect in healthy rats and analyzed nerve regeneration in poly (ε-caprolactone) (PCL) conduits longitudinally divided by gold (Au) and gold-cobalt oxide (AuCoO) nanoparticles embedded in poly-propylene poly-ethylene glycol (PPEG) membranes (AuPPEG or AuCoOPPEG) and compared it with unmodified PPEG-membrane and hollow PCL conduits. After 21 days, we detected significantly better axonal outgrowth, together with higher numbers of activated Schwann cells (ATF3-labelled) and higher HSP27 expression, in reconstructed sciatic nerve and in corresponding dorsal root ganglia (DRG) in the AuPPEG and AuCoOPPEG groups; whereas the number of apoptotic Schwann cells (cleaved caspase 3-labelled) was significantly lower. Furthermore, numbers of activated and apoptotic Schwann cells in the regenerative matrix correlated with axonal outgrowth, whereas HSP27 expression in the regenerative matrix and in DRGs did not show any correlation with axonal outgrowth. We conclude that gold and cobalt-oxide nanoparticle modified membranes in conduits improve axonal outgrowth and increase the regenerative performance of conduits after nerve reconstruction.

## 1. Introduction

Reconstruction of a peripheral nerve defect due to transection or laceration, in which the re-approximation of the injured nerve ends is not possible, could be a clinical challenge. If a primary suture is not feasible, appropriate interfascicular autologous nerve grafting of short and long defects is still the gold standard procedure [1]. Alternative techniques for clinical approaches, such as nerve conduits and nerve allografts [2], are also available [3,4]. Despite these advanced surgical techniques, treatment may still result in an incomplete restoration of motor and sensory function of the limb, severe disability for the patients and a socioeconomic burden to society [5]. To overcome this insufficiency, many different types of bioengineered nerve conduits have been introduced and described in experimental nerve injury models [6,7,8]. Bacterial polyesters [9], such as poly-3-hydroxyoctanoate [10] and poly-3-hydroxybutyrate [11], or biodegradable polymers, such as poly (ε-caprolactone) [12] or chitosan [13,14,15], have shown promising results. Poly (ε-caprolactone), a biodegradable polymer, is presented as an efficient alternative to autografts [16,17,18,19]. It is described as easy to handle, flexible and transparent, but somehow harder and more stable in the mechanical structure that overcomes chitosan conduits [16].

To further increase the regenerative capacity of a nerve conduit, one can modify the inner structure of the conduit, by simply longitudinally dividing the conduit into two chambers [20,21], administer cells [22] or apply growth factors [23]. Furthermore, recent studies introduced new generation nerve conduits that are enhanced with nanoparticles. Gold nanoparticles were shown to enhance nerve regeneration in rat sciatic nerve injury models and promote axonal outgrow [24,25,26]. They promoted adhesion and proliferation of Schwann cells in vitro with no toxic or immunogenic responses in vivo after 18 months of sciatic nerve injury and repair in rats [27]. Thus, gold nanoparticles at a membrane in a conduit may be an alternative to promote regeneration.

A magnetic nanoparticle, cobalt oxide, on the other hand, is known to express excellent catalytic, electrical and magnetic properties [28], and is used in cancer treatment, causing an increased amount of tissue degradation and apoptosis in tumor tissue [29]. Chitosan-cobalt oxide nanoparticles were found to activate cellular apoptosis by increasing cleaved caspase-3 activation in leukemic cells [30]. Cleaved caspase-3 activation in Schwann cells is known to be crucial in nerve regeneration after nerve injury [13,20,21]. Recently, cobalt -oxide nanoparticles were shown to enhance cancer cell death via targeting protein/organelle degradation pathways and inhibiting autophagia [31]. Gold-cobalt-oxide nanoparticle, instead, was also presented to have good catalytic property in polymeric reactions [32]. Hence, cobalt oxide particles, added to a gold nanoparticle membrane, may be a way to balance the proliferation of Schwann cells and possibly thereby the nerve regeneration.

In this present study, our aim was to investigate the regeneration capacity of poly (ε-caprolactone) (PCL) conduits by first; longitudinally dividing the conduit into two chambers by a poly-propylene polyethylene glycol (PPEG) membrane, and second; enhancing the membrane with gold (Au) or gold-cobalt-oxide (AuCoO) nanoparticles. We compared the axonal outgrowth in hollow conduit or conduit with unmodified or nanoparticles-modified membrane in a reconstruction of healthy rat sciatic nerve injury model after 21 days of implantation.

## 2. Results

### 2.1. SEM and EDS Analysis of the Membranes

**Figure 1:**
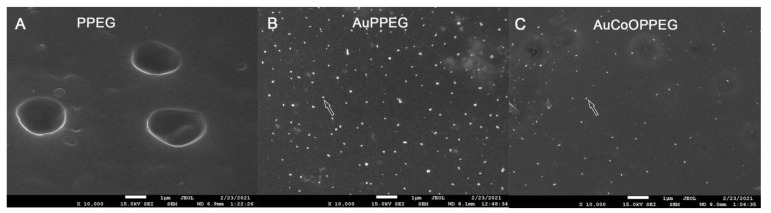
Scanning electron microscopy (SEM) analysis of the (**A**) Poly-propylene polyethylene glycol (PPEG) membrane, (**B**) Gold nanoparticle embedded PPEG membrane (AuPPEG) and (**C**) Gold and cobalt oxide nanoparticle embedded PPEG membranes (AuCoOPPEG) are presented. The pictures are taken under 10,000× magnification and white bar indicates 1 µm. The white nano-islands are thought to be gold or cobalt-oxide nanoparticles (arrows).

Scanning electron microscopy (SEM) analysis of the membranes was performed in order to document the presence of gold and cobalt-oxide nanoparticles on the PPEG membranes. PPEG membrane had no nanoparticle present on the surface, whereas, gold nanoparticle embedded PPEG membrane (AuPPEG) and gold-cobalt-oxide nanoparticle embedded PPEG membrane (AuCoOPPEG) had island-like structures on the surface, which were most probably gold and cobalt-oxide nanoparticles [32,33] (Figure 1). Energy dispersive X-ray spectroscopy (EDS) analysis of these sections confirmed the presence of the gold and cobalt-oxide nanoparticles (Supplementary data 1 with references [33,34,35,36,37]).

### 2.2. Macroscopic Analyses of the Regenerated Matrix 

A visible regenerative matrix was formed in all rats in both AuPPEG (10/10 rats) and AuCoOPPEG membrane groups (10/10 rats) followed by 8/10 rats in the PPEG membrane group and 6/10 rats in the hollow conduit group (Table 1). There was a statistical significance between groups in terms of thickness of regenerative matrix, expressed as none, loose or dense thickness, formed within the conduit (*p* = 0.015, Fisher’s exact test). The regenerative matrices were significantly thicker (>1 mm) in AuPPEG (6/10 rats) and AuCoOPPEG (6/10 rats) membrane groups compared to the hollow conduit group (Table 1). There was a statistical difference between groups in terms of number of cables formed within the conduit (*p* = 0.022, Fisher’s exact test), where two full length regenerative matrix cables were found in 6/10 rats in the AuCoOPPEG group, in 4/10 rats in the AuPPEG group and in 4/10 rats in the PPEG group (Table 1). The number of regenerated cables were significantly higher in the AuPPEG and AuCoOPPEG groups compared to the hollow conduit group (Table 1).

### 2.3. Immunohistochemical Analysis of the Thickest Cable of Regenerative Matrix

#### 2.3.1. Axonal Outgrowth

**Figure 2:**
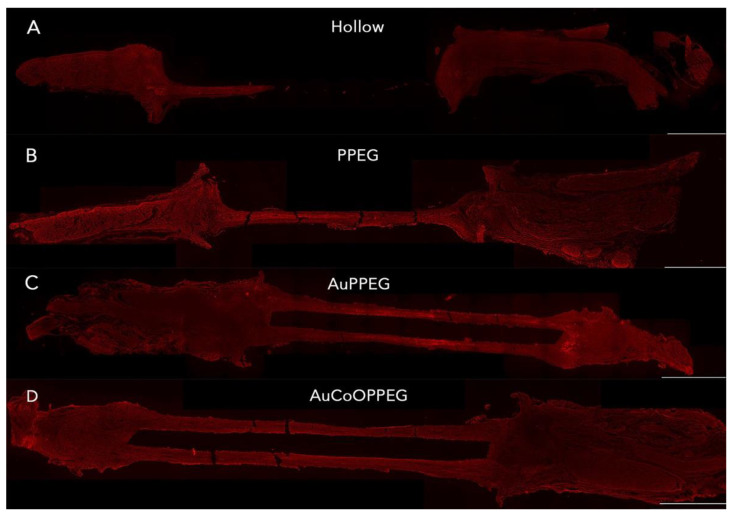
Axonal outgrowth presented as neurofilament staining in the experimental groups after reconstruction of 10 mm long sciatic nerve defects. The operated sciatic nerve with the proximal and distal nerve ends and the formed regenerated matrix are shown in (**A**) the hollow PCL conduit group, (**B**) PCL conduit with PPEG membrane group, (**C**) PCL conduit with AuPPEG membrane, (**D**) PCL conduit with AuCoOPPEG membrane. Bar = 2 mm.

The presence of neurofilament-positive axons indicated regenerating axons within the conduit. Regenerating axons seemed to bridge the nerve ends and were present in the distal nerve end in the majority of cases in AuPPEG group and AuCoOPPEG group (Figure 2, Table 2). With respect of length of axonal outgrowth, there was a statistical difference between groups (*p* = 0.016, Kruskal–Wallis, Table 2), with significantly longer axonal outgrowth in the AuCoOPPEG group than in the hollow conduit and PPEG membrane groups (Table 2).

#### 2.3.2. HSP27 Immunoreactivity

**Figure 3:**
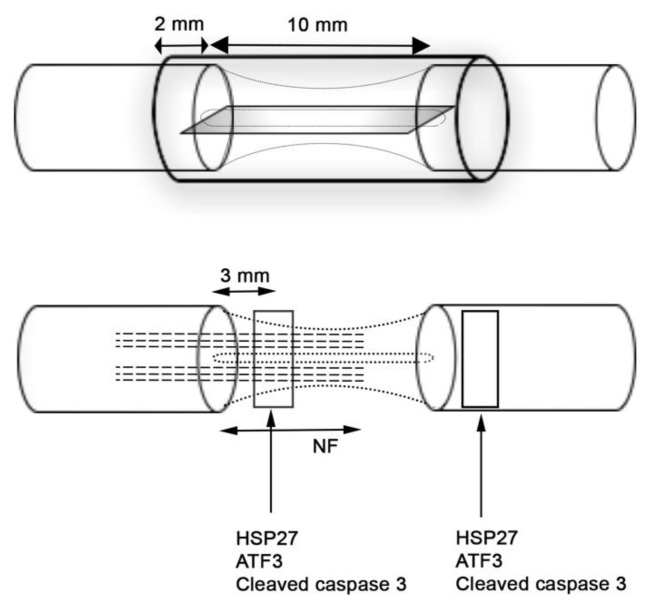
Schematic view of the conduit with inserted polymer membrane used to reconstruct a sciatic nerve defect with a final length of the defect of 10 mm. Nerve regeneration was evaluated at 21 days by immunohistochemistry for axonal outgrowth (length of neurofilaments; NF), presence of activated (ATF3-labelled) and apoptotic (cleaved caspase 3-labelled) Schwann cells as well as expression of HSP27 in the regenerative matrix (3 mm distal to proximal suture line) and in the distal nerve segment just after the distal suture line.

**Figure 4:**
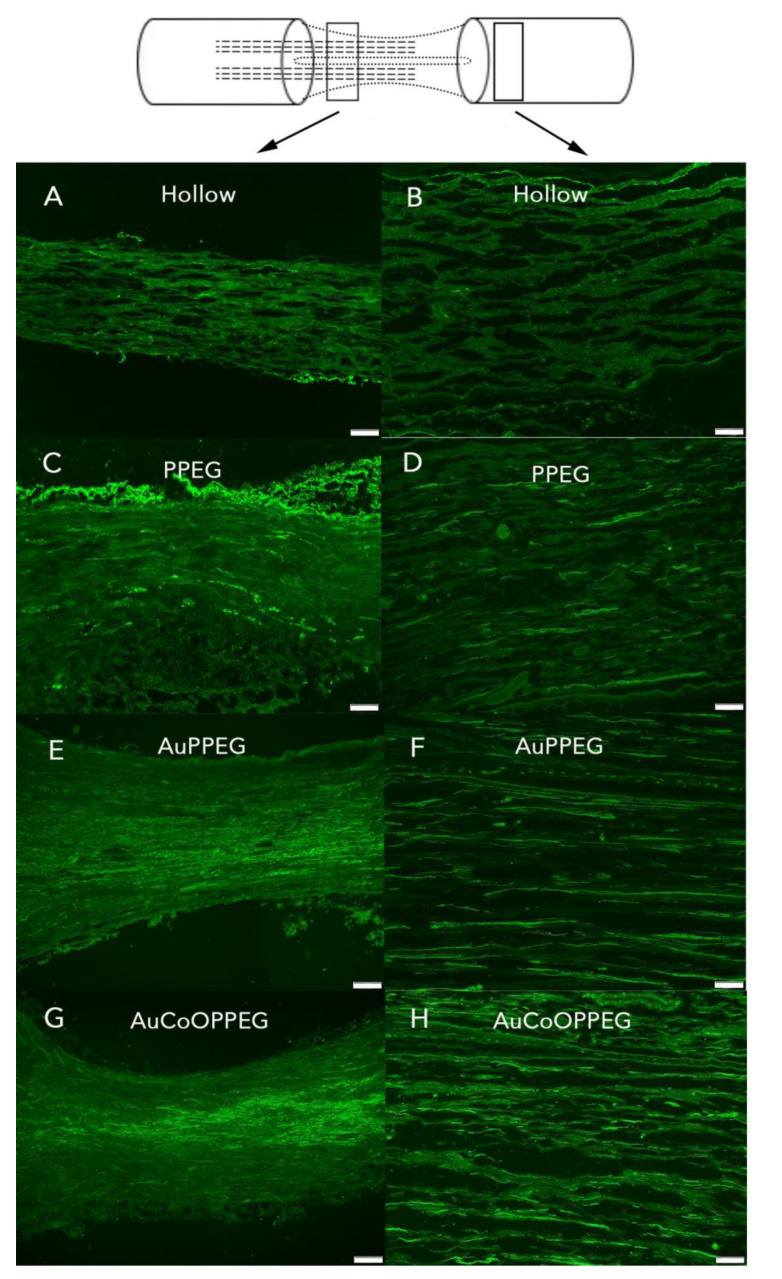
HSP27 immunoreactivity after reconstruction of a 10 mm long nerve defect in rat sciatic nerve is shown in the hollow PCL conduit group, (**A**) in proximal site, (**B**) in distal site; PCL conduit with PPEG membrane group, (**C**) proximal site, (**D**) distal site; PCL conduit with AuPPEG membrane, (**E**) proximal site, (**F**) distal site; and in the PCL conduit with AuCoOPPEG membrane, (**G**) proximal site, (**H**) distal site. The schematic drawing on the top indicates the proximal (3 mm distal to the proximal suture site—on the regenerated matrix) and distal site (just distal to the distal suture site) of the experimental sciatic nerve. Bar = 100 µm.

**Figure 5:**
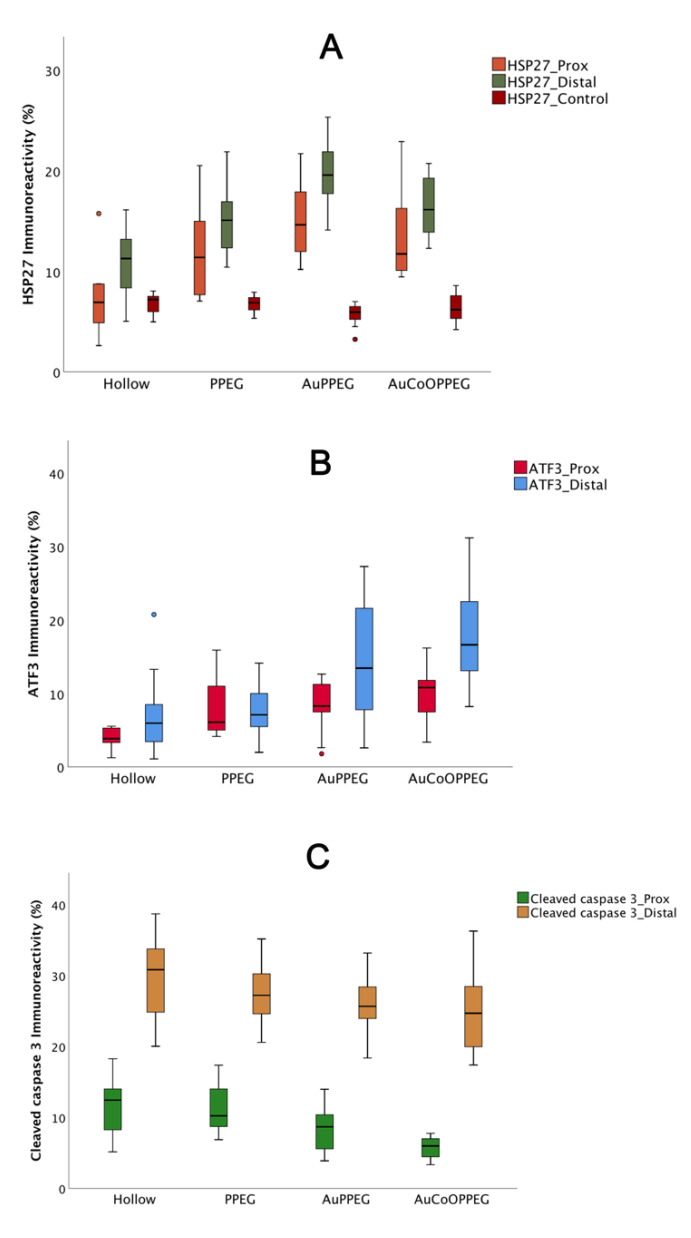
Boxplots of immunohistochemical analysis from the experimental and control sides in reconstructed rat sciatic nerves with different conduits at 3 mm distal to the proximal suture site in the regenerated matrix (Prox) and just distal to the distal suture site (Distal). The groups are coded according to the membrane properties, i.e., Hollow: hollow conduit, PPEG: PCL conduit with PPEG membrane, AuPPEG: PCL conduit with AuPPEG membrane, AuCoOPPEG: PCL conduit with AuCoOPPEG membrane. Box plots indicates the 25 and 75 percentiles (Tukey’s hinge) with the horizontal line in the middle indicating the median value. Error bars show minimum and maximum values. (**A**) HSP27 immunoreactivity data in both experimental and control sciatic nerves, (**B**) ATF3 immunoreactivity data in experimental sciatic nerve, (**C**) cleaved caspase 3 immunoreactivity data in experimental sciatic nerve.

HSP27 immunoreactivity was calculated on the thickest regenerated cable as the percentage of the total area on the nerve section proximal (3 mm distal to the proximal suture site in the regenerated matrix) and distally (just distal to the distal suture site) (Figure 3). The intensity differed among groups on both proximal and distal sites in the sciatic nerve (*p* = 0.023 proximally and *p* = 0.001 distally; Kruskal–Wallis, Figure 4). In the regenerated matrix (proximal site), the percentage of the HSP27 expression was significantly higher in the AuPPEG membrane and in the AuCoOPPEG groups compared to the hollow conduit group (Figure 5A, Table 2). Similarly, on the distal site, the percentage of the HSP27 immunointensity was significantly higher in both the AuPPEG and AuCoOPPEG groups compared to hollow group and PPEG group (Table 2 and Figure 5A). Additionally, the HSP27 immunoreactivity in distal injury site was significantly higher in the PPEG membrane group compared to hollow conduit group (Table 2). HSP27 was also expressed in control nerves with no statistical difference among groups (*p* = 0.15, Kruskal–Wallis; Table 2).

#### 2.3.3. ATF3 Immunoreactivity

**Figure 6:**
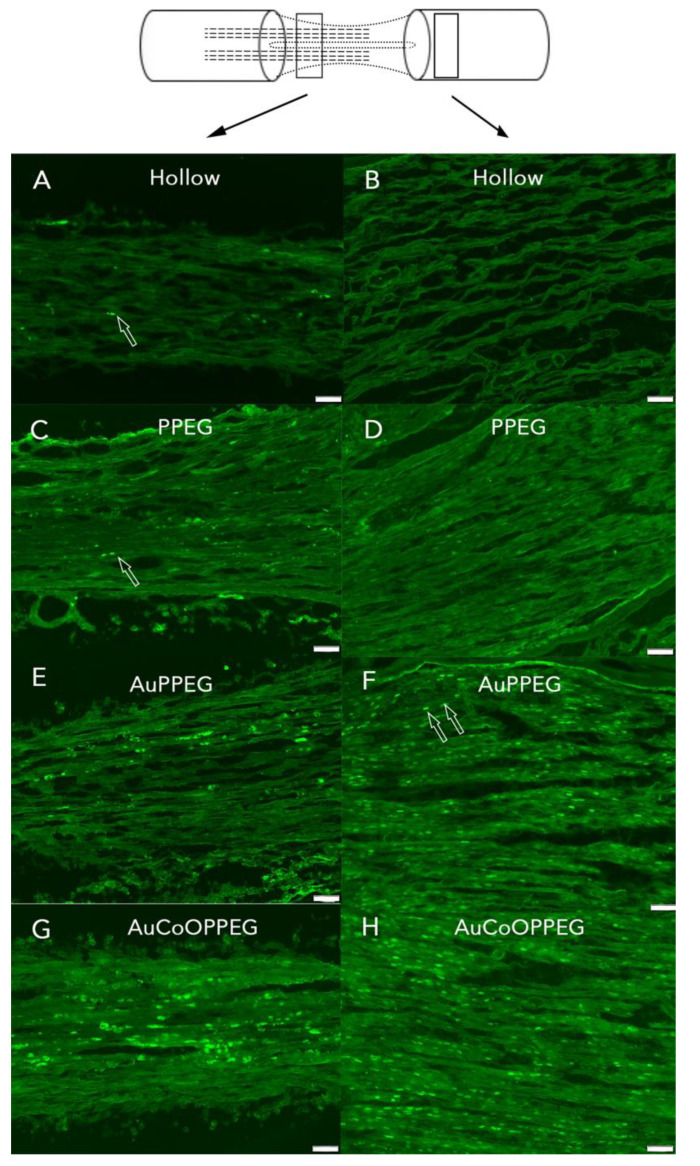
ATF3 immunoreactivity is shown in hollow conduits, (**A**) at proximal site, (**B**) at distal site; PCL conduit with PPEG membranes, (**C**) proximal site, (**D**) distal site; PCL conduit with AuPPEG membrane, (**E**) proximal site, (**F**) distal site; and in the PCL conduit with AuCoOPPEG membrane, (**G**) proximal site, (**H**) distal site. The schematic drawing on the top indicates the proximal (3 mm distal to the proximal suture site—on the regenerated matrix) and distal site (just distal to the distal suture site) of the experimental sciatic nerve. The arrows indicate ATF3-labelled Schwann cells. Bar = 50 µm.

In the sciatic nerve sections, cells with characteristics of a Schwann cell (long and oval nuclei), which was located longitudinally along the axon [21], were counted as ATF3 labelled cells (activated Schwann cells) (Figure 6). There were significant differences between groups with respect to ATF3 immunoreactivity at proximal site in the regenerating nerve (*p* = 0.022, Kruskal–Wallis, Table 2, Figure 5B). The conduits with AuCoOPPEG membranes revealed the highest percentage of activated Schwann cells, which was significantly higher than the hollow conduits (Table 2). Additionally, the conduits with AuPPEG and PPEG membranes had higher percentage of ATF3 immunoreactivity compared to hollow conduits (Table 2). In contrast, proximal nerve segments from the contralateral, uninjured, sciatic nerve, exhibited only a few positive ATF3-labelled cells in accordance with previous studies [38].

At the distal nerve site, there was a significant difference between groups with respect to ATF3 immunoreactivity (*p* = 0.002, KW, Table 2). The conduits with AuCoOPPEG membranes showed the highest percentage of activated Schwann cells with a significant difference compared to hollow conduits and conduits with PPEG membranes (Table 2). The percentage of activated Schwann cells were higher in conduits with AuPPEG membranes compared to hollow conduits (Table 2). Distal nerve segments from the contralateral, uninjured, sciatic nerve, exhibited only a few ATF3-labelled cells.

#### 2.3.4. Cleaved Caspase 3 Immunoreactivity

**Figure 7:**
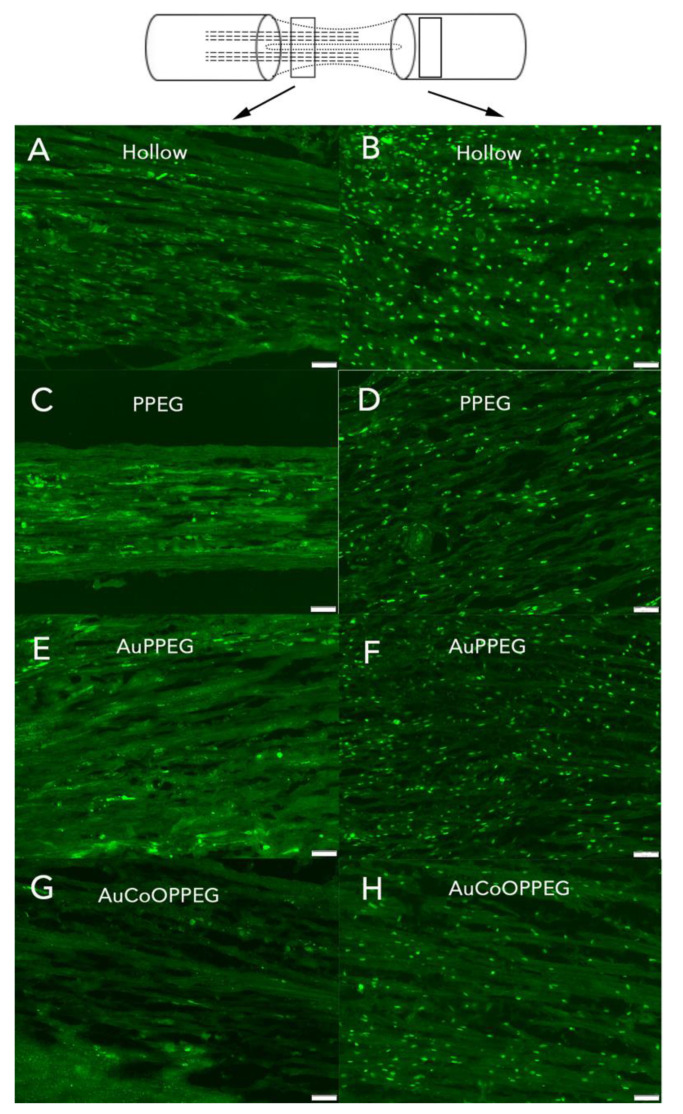
Cleaved caspase 3 immunoreactivity is shown in hollow PCL conduits, (**A**) at proximal site, (**B**) at distal site; PCL conduit with PPEG membrane, (**C**) proximal site, (**D**) distal site; PCL conduit with AuPPEG membrane, (**E**) proximal site, (**F**) distal site; and PCL conduit with AuCoOPPEG membrane, (**G**) proximal site, (**H**) distal site. The schematic drawing on the top indicates the proximal (3 mm distal to the proximal suture site—in the regenerated matrix) and distal site (just distal to the distal suture site) of the experimental sciatic nerve. The arrows indicate the cleaved caspase 3-(labelled Schwann cells. Bar = 50 µm.

There was a statistical difference between groups with respect to cleaved caspase 3 immunoreactivity (the percentage of apoptotic Schwann cells) in the regenerated matrix (*p* = 0.004, KW, Table 2, Figure 5C). The percentage of cleaved caspase 3 immunoreactivity tended to decline from higher to lower values from hollow conduits to conduits with AuCoOPPEG membranes (Figure 7). AuCoOPPEG presented the lowest percentage of apoptotic Schwann cells among all groups, being statistically significant compared to the other three groups (Table 2). At the distal nerve site a similar declining tendency was observed, but without any significant difference (*p* = 0.23, KW, Table 2). The contralateral unoperated sciatic nerve revealed no cleaved caspase 3 immunoreactivity both at the proximal and distal nerve sites.

#### 2.3.5. DAPI Positive Cells

In the regenerated matrix, the total number of DAPI positive cells did not differ statistically between groups (*p* = 0.38, KW, Table 2). However, the number of DAPI positive cells differed statistically at the distal site of the nerve (*p* = 0.03, KW, Table 2), being higher in the group with conduits with AuCoOPPEG membranes compared to hollow conduits and conduits with PPEG membranes (Table 2).

### 2.4. Immunohistochemical Analysis of the Thinner Cable of Regenerative Matrix

In several cases among the various groups, two regenerated cables were detected within the conduit: main regenerated cable, i.e., thicker cable (diameter measured in the midway at the time of analysis) and a second, thinner cable. Analyses of the second cables showed that AuCoOPPEG membrane-conduits revealed the longest axonal outgrowth and highest number of activated Schwann cells (Table 3). HSP27 expression was highest in conduits with AuPPEG membranes. The percentage of cleaved caspase 3-labelled Schwann cells, on the other hand, was lower in both AuPPEG and AuCoOPPEG membrane groups. This pattern of immunoreactivity resembles the immunoreactivity of the main-thickest regenerated cable presented in Table 2.

Axonal outgrowth is presented as the NF immunoreactivity. The intensity of the HSP27 immunoreactivity is presented as the percentage of the total area (%). The numbers of ATF3 and cleaved caspase 3-labelled Schwann cells are expressed in percentage of the total number DAPI positive cells at the 3 mm proximal site of the regenerated matrix. The results are presented as median (minimum and maximum). Statistical analysis is not performed due to limited number of observations.

### 2.5. Immunohistochemical Analysis of DRG 

**Figure 8:**
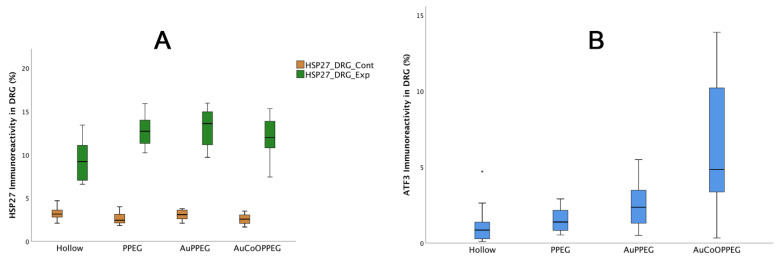
Boxplots of HSP27 and ATF3 immunoreactivity of dorsal root ganglia (DRG) after nerve reconstruction of a 10 mm long nerve defect in rat sciatic nerve is presented. The groups are coded according to the membrane properties, i.e., Hollow: hollow conduit, PPEG: PCL conduit with PPEG membrane, AuPPEG: PCL conduit with AuPPEG membrane, AuCoOPPEG: PCL conduit with AuCoOPPEG membrane. Box plots indicate the 25 and 75 percentiles (Tukey’s Hinge) with the horizontal line in the middle indicating the median value. Error bars show min-max values. (**A**) HSP27 immunoreactivity data in experimental and unoperated (control) side DRGs, (**B**) ATF3 immunoreactivity data in experimental DRGs. Cont: DRGs on the unoperated-control side, Exp: DRGs on the experimental side.

**Figure 9:**
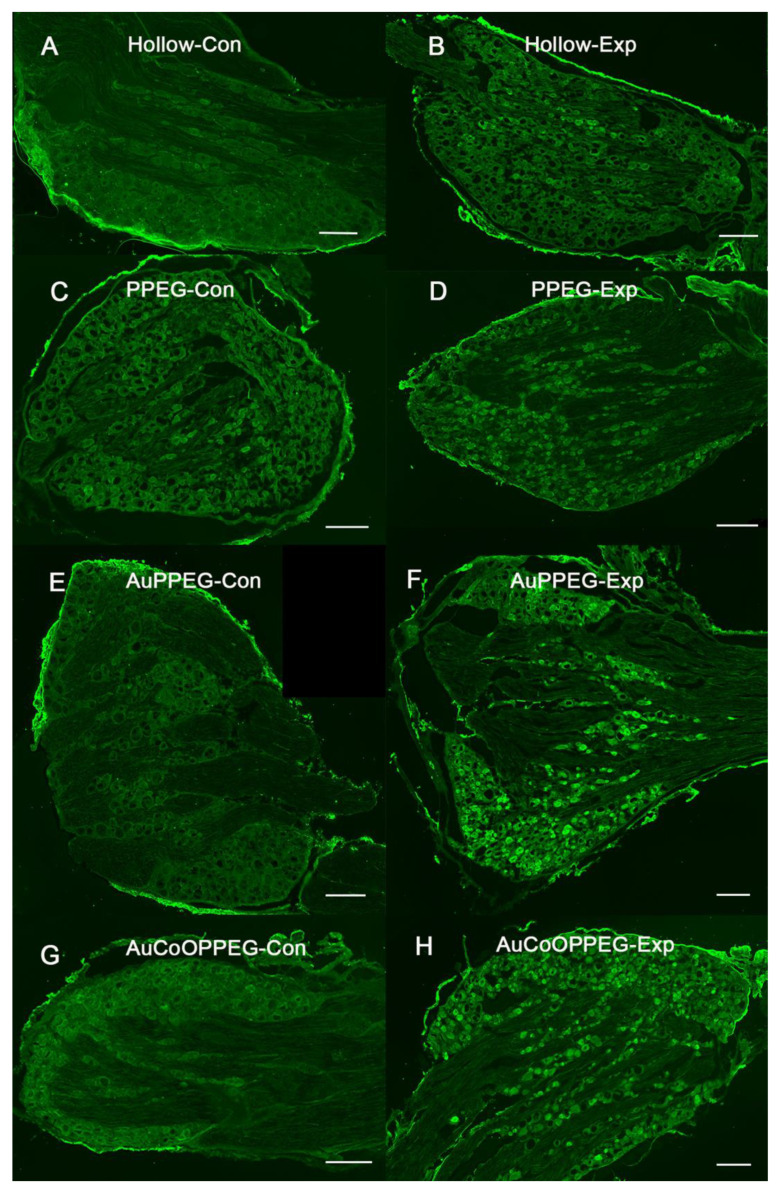
HSP27 immunoreactivity in whole DRG in hollow PCL conduit group, (**A**) control, (**B**) experimental; PCL conduit with PPEG membrane, (**C**) control, (**D**) experimental; PCL conduit with AuPPEG membrane, (**E**) control, (**F**) experimental; and in PCL conduit with AuCoOPPEG membrane, (**G**) control, (**H**) experimental. Bar = 200 µm.

HSP27 expression was observed in control dorsal root ganglia (DRG) side at around 3% of the total area of the ganglion with no significant differences between groups (*p* = 0.18, KW, Table 4, Figure 8A, Figure 9). The percentage of HSP27 immunoreactivity in DRG at the experimental side differed significantly between groups (*p* = 0.005, KW, Table 4). The expression of HSP27 in DRG’s was significantly higher in the conduits with PPEG, AuPPEG and AuCoOPPEG membranes compared to hollow conduits (Table 4). Similarly, the HSP27 ratio (the ratio of HSP27 immunoreactivity in the experimental DRG to the control DRG) also differed statistically (*p* = 0.015, KW), being significantly higher in the conduits with PPEG, AuPEG and AuCoOPPEG membranes compared to hollow conduits (Table 4).

**Figure 10:**
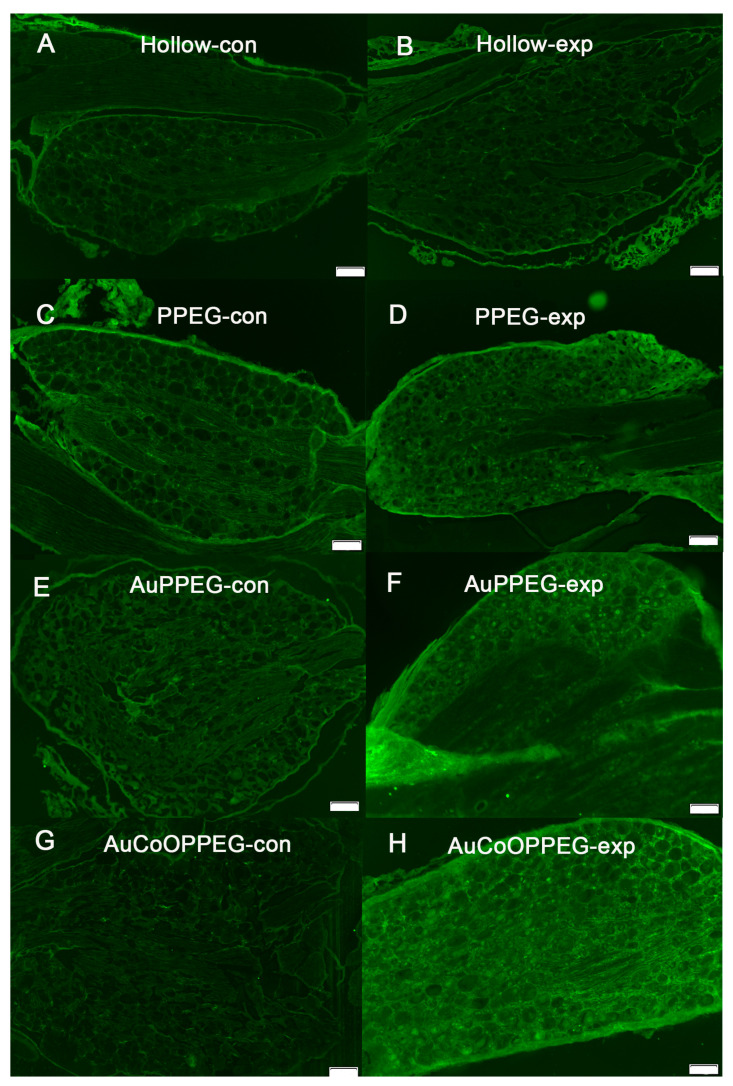
ATF3 immunoreactivity in whole DRG in hollow PCL conduits, (**A**) control, (**B**) experimental; PCL conduit with PPEG membrane, (**C**) control, (**D**) experimental; PCL conduit with AuPPEG membrane, (**E**) control, (**F**) experimental; and in PCL conduit with AuCoOPPEG membrane, (**G**) control, (**H**) experimental. Bar = 100 µm.

The sensory neurons from the control side did not show any staining for ATF3, while the experimental side showed ATF3 staining; difference being significant (*p* = 0.002, KW, Table 4, Figure 8B, Figure 10). The number of ATF3-labelled sensory neurons was significantly higher in conduits with AuCoOPPEG membranes compared to hollow conduits and conduits with PPEG membranes (Table 4). The conduits with AuPPEG membranes had significantly higher number of ATF3-labelled sensory neurons compared to hollow conduits (Table 4).

### 2.6. Correlation and Regression Analyses

**Figure 11:**
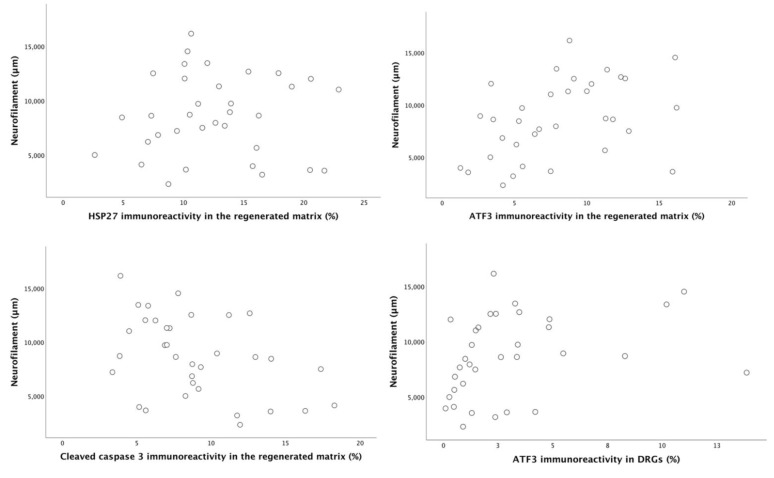
Scatter plots for axonal outgrowth (neurofilament staining in µm) against the percentage of HSP27 immunoreactivity, percentage of ATF3 and cleaved caspase 3 in the regenerated matrix and the percentage of ATF3 immunoreactivity in DRG after reconstruction of a 10 mm nerve defect in rat sciatic nerve is presented. For r- and *p*-values see the results section.

Correlation and regression analyses were used to evaluate the association between axonal outgrowth and activated and apoptotic Schwann cells as well as expression of HSP27 at the experimental sciatic nerve and in the DRGs. There were no significant correlations between the evaluated variables when the four different groups were analyzed individually, probably due to a limited number of observations in the individual groups. When analyzing all the treatments grouped together, as pooled data, axonal outgrowth showed a positive correlation with the percentage of ATF3- labelled Schwann cells (r = 0.47, *p* = 0.005, Spearman correlation) and a negative correlation with cleaved caspase 3-labelled (r = –0.42, *p* = 0.013, Spearman correlation) Schwann cells in the regenerated matrix. Furthermore, axonal outgrowth correlated with percentage of activated Schwann cells (ATF3- labelled) in DRG (r = 0.46, *p* = 0.006, Spearman correlation; Figure 11). There was no correlation between axonal outgrowth and the expression of HSP27 in the regenerated matrix (r = 0.06, *p* = 0.72, Spearman correlation) and in the experimental DRG (r = 0.34, *p* = 0.053, Spearman correlation).

A regression analysis was used to primarily evaluate the association between axonal outgrowth (dependent factor) and activated and apoptotic Schwann cells as well as expression of HSP27 in experimental sciatic nerve and DRG. Our second purpose for using regression analysis was to see the impact of different types of membranes on the degree of axonal outgrowth. The simple linear regression showed that the presence of membrane in a conduit causes nearly 4000 µm longer axonal outgrowth than being a hollow conduit (unstandardized Beta 3931 (95% CI (792–7071)); *p* = 0.016). Similarly, the presence of gold and cobalt-oxide nanoparticles causes nearly 3000 µm increase in length of axonal outgrowth than having a plain or no membrane in the conduit (unstandardized Beta 3120 (95% CI (468–5773)); *p* = 0.023). The simple linear regression showed that an increase in percentage of activated Schwann cells in the regenerative matrix had a positive impact on axonal outgrowth (unstandardized Beta 370 (95% CI (77–663)); *p* = 0.015). On the other hand, an increase in percentage of apoptotic Schwann cells in the regenerative matrix had a negative impact on axonal outgrowth unstandardized Beta −370 (95% CI (−713 to −99)); *p* = 0.011). The percentage of activated Schwann cells (ATF3-labelled) in DRG and the percentage of HSP27 expression in both the regenerated matrix and in DRG had no impact on axonal outgrowth (*p* = 0.06, *p* = 0.80, *p* = 0.73, respectively).

## 3. Discussion

In this study, we investigated the local neuroregenerative effect of unmodified and modified PPEG membranes with gold and gold-cobalt-oxide nanoparticles in PCL conduits and compared them with hollow PCL conduits bridging a 10 mm rat sciatic nerve defect. Our data pointed out that a PCL conduit with a AuPPEG or a AuCoOPPEG membrane had a better nerve regeneration with longer axonal outgrowth after 21 days of implantation, which is a suitable time point to evaluate nerve regeneration in conduits in order to get an indication of efficacy of such conduits. These nanoparticle-modified conduit groups revealed significantly higher amounts of activated Schwann cells and lower amounts of apoptotic Schwann cells together with higher HSP27 expression in regenerative matrix. The presence of activated and apoptotic Schwann cells in the regenerative matrix was found to be correlated to and have a direct impact on the axonal outgrowth. Interestingly, however, the DRG response with higher ATF3- labelled sensory neurons and higher HSP27 expression in sensory neurons and in regenerative matrix was found to have no influence on the axonal outgrowth.

Previous studies showed that axonal outgrowth after reconstruction of the nerve defect is closely related to interactions between activated and apoptotic Schwann cells existing in the microenvironment on both proximal and distal sites of the lesion [39,40,41,42]. It is also known that sensory neurons in DRG are affected by injury and respond to nerve regeneration [38]. In our study, we used gold and both gold and cobalt oxide nanoparticles to enhance the PPEG membrane. In the conduits with nanoparticle embedded membranes, a thicker regenerative matrix, with one or two cables, was formed and outgrowing axons successfully bridged the nerve defect after 21 days of surgery. Additionally, activated Schwan cells (i.e., ATF3-labelled), which are responsible for attracting the regrowing axons [39] displayed in significantly higher amounts in both the regenerated nerve and in the distal nerve segment. Simultaneously, the apoptotic events, as indicated by cleaved caspase 3- labelled Schwann cells, were reduced in the regenerated segments in conduits with nanoparticle embedded membranes, indicating that events potentially deleterious for axonal regeneration were reduced. Additionally, HSP27, known as a neuroprotective factor by decreasing cellular stress [43,44,45], was found to be significantly higher in these groups, both in the proximal regenerated nerve and the distal nerve segment, but had no direct impact on axonal outgrowth as revealed by the regression analysis [46]. The support for nerve regeneration, most probably by any neuroprotective mechanism(s) [47], in conduits with nanoparticle embedded membrane is further strengthened by the higher number of activated sensory DRG neurons (i.e., ATF3 labelled) and higher percentages of the HSP27 expression in DRG.

A recent study showed that a nerve injury leads to increased levels of caspase-3 mRNA and active caspase-3 protein in the DRG [48]. HSP27, a molecule that combats cellular stress [43,44,45], is known to be expressed after peripheral nerve injury in the sensory neurons in DRG and on the axonal growth cone [49]. However, its interaction with the expression of ATF3 and cleaved caspase 3 at the proximal and distal injury site is not well described. With our study, we aimed to see the association between axonal outgrowth and activity of Schwann cells and the expression of HSP27 in the nerve injury sites and the response of sensory neurons in DRG. We found that axonal outgrowth positively correlated with ATF3 immunoreactivity both in the proximal injury site and in the DRG and negatively correlated with cleaved caspase 3 immunoreactivity at the proximal injury site. Interestingly, no association was found with expression of HSP27 on the proximal lesion site, while the presence of activated and apoptotic Schwann cells in the proximal site had direct impact on length of axonal outgrowth, as shown in regression analysis. Furthermore; even though the ATF3 immunoreactivity in DRG was found to be positively correlated to axonal outgrowth, it had no direct influence on axonal outgrowth, according to regression analysis. In other words, activated and apoptotic Schwann cells in regenerated matrices play an active role in axonal outgrowth, whereas ATF3 immunoreactivity in DRG and HSP27 expression in both reconstructed nerve and in DRGs may act as neuroprotective molecules, rather than directly acting on axonal outgrowth [47].

Besides the role of Schwann cells in peripheral nerve regeneration, it is also known that the inflammatory response, conducted by macrophages in both DRGs and injured nerve ends after peripheral nerve injury, known as neuroinflammation, has both a beneficial and a negative effect on axonal outgrowth [50]. As a limitation in our study, we did not analyze the inflammatory response which is known to play an active role in nerve regeneration.

Many different types of bioengineered nerve conduits have been introduced in experimental nerve injury models, after initial presentation of the clinical usefulness of conduits in nerve repair [51], pointing out the need for improving the inner surface of the conduit [52,53,54]. Such a surface can structurally be modified by adding grooves [55] or pores [56], or by simply dividing it into multiple channels [43]. In our study, we used polypropylene polyethylene glycol (PPEG) co-polymer as a membrane structure to achieve a two chambered conduit and showed that a PPEG membrane, especially with nanoparticles embedded, leads to a longer axonal outgrowth at 21 days with signs of better neuroprotection compared to that of hollow conduits, i.e., higher HSP27 expression. Polyethylene glycol (PEG) is known as a membrane sealant and by silencing oxidative stress on neurons, is known to be neuroprotective [57]. In our previous studies, we showed that PPEG is a biocompatible, highly soluble copolymer and suitable for nanoparticle application due to its stable structure [58,59]. Different types of nerve conduits with PEG [60,61] have revealed successful axonal outgrowth in rat models after a sciatic nerve injury. PEG-coated Au nanoparticles have been applied in a mouse spinal cord injury model, showing functional recovery, attenuation of microglial response, and increased remyelination at eight weeks after treatment [57]. Our study showed that a PPEG membrane in a conduit seemed to support the formation of a regenerative matrix creating a full-length regenerated cable with axonal outgrowth within the conduit. Furthermore, we showed that the second cable that is formed within the conduits mimics the immunohistochemical expression of the main regenerated cable. This could be explained by the fact that a highly soluble PPEG membrane enhances the cellular interactions and permits communication between the two compartments of the conduit.

Nanoparticles became the choice of modification due to their inert and non-immunogenic characteristics, good biocompatibility, relatively low toxicity and wide range of possible surface functionalization [24,26,27,62]. Baranes et al. reported an enhanced neurite outgrowth with electrospun nanofibers, which were coated by Au nanoparticles [25]. Another nerve guide fabricated by adsorbing Au nanoparticles onto silk fibers has been found to promote adhesion and proliferation of Schwann cells in vitro with no toxicity in a rat sciatic nerve over a period of 18 months [27]. Furthermore, another in vitro study showed that Au nanoparticles increased the conductivity of the scaffold and lead to increased biological response of the Schwann cells [63]. A recent study demonstrated that a continuous secretion of gold nanoparticles, combined with neurotrophic factors, with adipose-derived stem cells enhanced nerve regeneration and myelination [24]. These studies speculated that changes in intracellular signalling pathways were responsible for nerve regeneration and outgrowth of axons and suggested that the process is influenced by the mechanical support of the guides with gold nanoparticles. In our study, we used gold and both gold and cobalt-oxide nanoparticles to enhance the PPEG membrane. In the conduits with nanoparticle embedded membranes, a thicker regenerative matrix, with one or two cables, was formed and outgrowing axons successfully bridged the nerve defect after 21 days of surgery. Gold and gold-cobalt nanoparticle-modified conduit groups revealed significantly higher amounts of activated Schwann cells and lower amounts of apoptotic Schwann cells together with higher HSP27 expression in regenerative matrices. Higher numbers of activated sensory DRG neurons (i.e., ATF3 -labelled) and higher percentages of HSP27 expression in DRG was present in both gold and gold-cobalt nanoparticle-modified conduits.

Cobalt-oxide nanoparticle, a magnetic metal nanoparticle [32], has been shown to act as a functional anticancer agent and drug delivery vehicle [31]. Chitosan-cobalt-oxide nanoparticles were found to activate cellular apoptosis by increasing cleaved caspase 3 activation in leukemic cells [30]. It is reported that the apoptotic and cytotoxic actions of the cobalt-oxide nanoparticles occur after intracellular uptake of the cobalt-oxide nanoparticles [30,31]. In contrast, we detected a decrease in cleaved caspase 3-labelled Schwann cells in conduits with AuCoOPPEG membranes with an enhanced axonal outgrowth in such a supportive regenerative microenvironment. This effect could be due to a local extracellular activity of cobalt-oxide nanoparticles acting synergistically with gold nanoparticles. Gold and cobalt-oxide nanoparticles could possibly form a nanoisland on the PPEG membrane, causing an external surface change to the membrane. This surface becomes easily detectable by the Schwann cells in the regenerative matrix within the conduit and by this way axonal outgrowth could be enhanced [54].

Although medical applications of nanoparticles are expanding, cellular toxicity is presented to be a handicap in in vivo studies. It is known that high concentration of metal nanoparticles in living organisms can cause cell oxidative stress and reactive oxygen species production, leading to cell membrane disruption, DNA damage and cell death [62]. Significantly higher numbers of apoptotic and oxidatively-stressed cells have been detected after gold nanoparticle exposure to mouse retina [64] or to NG108-15 neuronal cells [62]. Cobalt-oxide nanoparticles, on the other hand, activate protein degradation and apoptosis and cause cellular toxicity after intracellular uptake in treated cancer cells, but were found to be non-toxic to normal leucocytes at up to 100 µg/mL [31]. The toxicity of metal nanoparticles is known to be due to two factors: first, intracellular, or more precisely intranuclear, activity of the nanoparticle and second; the degree of the release of the nanoparticles from the nanocomplex [30]. It is presented that these effects can be minimized: first, by using lower concentrations of nanoparticles, second, by using larger particles than about 15 nm and third, by integrating nanoparticles to polymers, such as PEG or chitosan, which creates a more stable base-structure for nanoparticle [62]. In our study, the size of the gold and cobalt-oxide nanoparticles were greater than 50 nm (around 70–100 nm), and they were embedded into PPEG membranes, which probably increased the stability of the nanoparticles. Furthermore, we did not detect any trace of nanoparticles systemically (scanning electron microscopy and energy dispersive X-ray spectroscopy analysis for gold and cobalt-oxide nanoparticles in rat spleen and liver tissues) after 21 days of the implantation (Supplementary data 2). We speculate that nanoparticles within the PPEG membrane were anchored into the polymer and became more stable. Therefore, they cannot be taken intracellularly and act only locally in the regenerative microenvironment.

## 4. Materials and Method

### 4.1. Pilot Study with Different Types of Nanoparticles and Membranes

To see the effect of different membrane structures on nerve regeneration, we conducted a pilot study on 10 and 15 mm long sciatic nerve defect model in rats with different types of nano-structured membranes in PCL conduits (Supplementary file 3). We used four different nanoparticle-modified amphiphilic copolymers as membranes with gold, silver and a mixture of gold and cobalt-oxide nanoparticles embedded into them: silver nanoparticle embedded poly-propylene polyethylene glycol (AgPPEG), gold nanoparticle embedded poly (N-izopropyl acryl amid-poly(3-hydroxy undecenoate) (AuPHU-PNIPAM), gold nanoparticle-embedded poly propylene poly-ethylene glycol (AuPPEG), and gold-cobalt-oxide nanoparticle-embedded poly-propylene poly-ethylene glycol (AuCoOPPEG). Among those different conduits, we detected longer axonal outgrowth with a complete regenerative matrix cable in a 15 mm nerve gap model and two complete regenerative matrix cables in 10 mm nerve gap model in both gold and gold-cobalt-oxide nanoparticle-embedded polypropylene polyethylene glycol membrane groups compared to other types of membranes (Supplementary file 3). This indicated a possible superior local nerve regeneration effect of both gold and gold-cobalt-oxide nanoparticles. With this information, we decided to further analyze the effect of these nanoparticles on nerve regeneration in modified conduits in the present study.

### 4.2. Production of Poly (ε-caprolactone) (PCL) Conduits with Modified Membranes

All the chemical materials used in production of the conduits were supplied from Sigma-Aldrich Chemical company, St. Louis, MO, USA. Tetra hydro furan (THF) was passed through a neutral Al_2_O_3_ column before use. PCL was supplied in granule form with Mn 80,000 g/mol. Chlorinated polypropylene (PP-Cl) has one chlorine atom on average in three repeating units with MW 147 Da. Polyethylene glycol was supplied in MW 2000 Da (PEG-2000). NaH was supplied as 60wt% in oil.

#### 4.2.1. Preparation of Poly (*ε*-caprolactone) (PCL) Conduits

Tetra hydro furan (THF) was passed through a neutral Al_2_O_3_ column before use. The amount of 5.84 g of PCL was dissolved in THF (60 mL) in a Pyrex test tube (Ø = 14 mm, length = 120 mm). The stainless steel Kirshner wire rods (*n* = 6, Ø = 2 mm, length = 300 mm) were dipped into the solution 20 times. After each dipping, the rod was kept outside for 5 min in order for the solvent to be evaporated. Finally, the rod was coated with PCL layer by layer in this way. The obtained PCL conduit was taken out from the stainless-steel rod and cut into 14 mm long (0.5 mm wall thickness and 2 mm in diameter) conduits.

#### 4.2.2. Synthesis of Polypropylene-Polyethylene Glycol (PP-g-PEG) Amphiphilic Copolymer

The Williamson-ether-synthesis-like reaction between PEG and PP-Cl was performed according to previously reported methods with slight modifications [65]. Briefly, PP-Cl (5.72 g, 0.040 mmol; 38 mmol Cl) was dissolved in 50 mL of THF (solution A) in a 500 mL bottle. PEG-2000 (7.31 g, 3.65 mmol) was dissolved in dry THF (100 mL). To this solution, 0.87 g, 22 mmol of NaH was added portion wise, while stirring continuously. After half an hour, the sodium salt of the PEG solution was poured into the PP-Cl solution (solution A) during continuous stirring. When the color of the solution mixture became yellow-brown after 40 min, the reaction was stopped by adding 5 mL of methanol. This solution was then poured into 500 mL of distilled water containing 5 mL of concentrated HCl, while stirring continuously. The crude polymer was taken and soaked in fresh distilled water (500 mL) for 24 h. The polymer was taken out of the distilled water, and dried in air at room temperature for 24 h. The obtained PP-g-PEG (PPEG) amphiphilic polymer was then dried under vacuum for 48 h at room temperature. At the end of the process, the yield polymer weight was 5.65 g.

#### 4.2.3. Synthesis of Gold Nanoparticle-Embedded PP-g-PEG Amphiphilic Graft Copolymers

Au-PP-g-PEG (AuPPEG) nanocomposite was prepared similar to a previous report [58]. The PP-g-PEG graft copolymer (0.81 g) was dissolved in 40 mL of THF. To this solution was added 0.020 g of HAuCl4, while continuously stirring for 20 min. Furthermore, 0.036 g of NaBH4 in 0.100 g of distilled water was added to this mixture, under continues stirring. Over the next 30 min, the solution turned violet.

#### 4.2.4. Synthesis of Gold and Cobalt-Oxide Nanoparticle-Embedded PP-g-PEG Amphiphilic Graft Copolymers

Au-CoO-PP-g-PEG (AuCoOPPEG) nanocomposite was prepared similar to a previous report with slight modifications [32,33]. A typical procedure is as follows: the PP-g-PEG (PPEG) graft copolymer (0.81 g) was dissolved in 40 mL of THF. To this solution, 0.020 g of the HAuCl_4_ and 0.050 g of aqueous solution of CoCl_2_.6H_2_O (0.025 g of CoCl_2_.6H_2_O in 0.025 g of distilled water) was added, while continuously stirring for 20 min. Furthermore, 0.036 g of NaBH_4_ in 0.100 g of distilled water was added to this mixture during continuous stirring. Over the next 30 min, the solution turned a deep blue-violet. 

#### 4.2.5. Modified Nano-Composite PCL Conduit Formation

Polymer membranes were prepared using solvent casting from THF [66]. The THF solution of the polymer (PPEG, AuPPEG or AuCoOPPEG, 0.87 g in 20 mL of THF) was filtered into a Petri dish (diameter = 5 cm) and the solvent was allowed to evaporate leaving a thin polymer membrane. The solvent cast membrane was submersed into distilled water (200 mL) for 24 h. Then, the polymer membrane was dried in air at room temperature for 24 h. The air-dried polymer membrane was dried under vacuum for 48 h for further drying. From each polymer membrane, 0.1 mm thick, 2 mm width and 10 mm length rectangular membranes were cut (*n* = 10 for PPEG membrane, *n* = 10 for gold nanopolymer membrane (AuPPEG) and *n* = 10 gold-cobalt-oxide nanopolymer membrane (AuCoOPPEG)) and placed into the conduits. Finally, nano-conduits were formed with 14 mm long hollow PCL conduits with or without 10 mm long nanocomposite membrane placed manually parallel to the long axis of the conduit allowing insertion of 2 mm proximal and distal nerve ends in order to apply and secure their positions with sutures (Figure 3). The position of the membrane was checked before performing the surgery. The surgical method, by having both the proximal and distal nerve ends inserted by 2 mm into the conduit, attached the membrane to both the nerve ends as well as anchored the nerve ends by sutures to the wall of the conduit resulting in an optimal fixation and position. 

### 4.3. Scanning Electron Microscopy (SEM) and Energy-Dispersive X-ray Spectrometry (EDS) Analysis of the Membranes

In order to document nanoparticles on the membranes, scanning electron microscopy (SEM) and energy dispersive X-ray spectroscopy (EDS) was performed. One sample from each membrane, i.e., PPEG membrane, AuPPEG membrane and AuCoOPPEG membrane were taken and mounted on metal stubs with a double-sided adhesive band. Only the PPEG membrane specimen was coated with gold (Emitech K550X Sputter Coating Systems, Quorum Technologies, UK). All samples were examined under a scanning electron microscopy (SEM) (Jeol JSM-7600F, Joel Ltd., Tokyo, Japan) at an accelerating voltage of 15 kV. On the same locations, elemental analysis with semi quantitative-Inca-energy dispersive X-ray spectroscopy (EDS) was performed.

### 4.4. Animals

A total of 40 female Wistar (Taconic, Denmark) rats (~200 g each) were included. All animals had a 12-h day–night cycle in their cages. Before surgery, the rats were anesthetized with an intraperitoneal injection of a mixture of Rompun (20 mg/mL; Bayer Health Care, Leverkusen, Germany) and Ketalar (10 mg/mL; Pfizer, Helsinki, Finland) with a dose of 1 mL Ketalar and 0.25 mL Rompun per 100 g body weight rat. For pain relief, all rats were postoperatively injected with Temgesic 0.01–0.05 mg/kg (0.3 mg/mL; Schering-Plough Europe, Brussels, Belgium).

### 4.5. Surgery

The sciatic nerve was exposed at the hind-limb level unilaterally and a 5 mm segment of the nerve was removed. A 14 mm long designed conduit was placed within the nerve defect and 2 mm of the proximal and distal ends each of the nerve was inserted into the conduit (Figure 3). Then, the conduit was sutured to the epi-/perineurium using a single suture of 9–0 Ethilon (Ethicon, Johnson & Johnson, Livingston, UK). With this form of placement, the resulting nerve defect within the conduit becomes 10 mm. The following conduits were used: (a) hollow PCL conduit (*n* = 10); (b) PCL conduit with PPEG membrane (*n* = 10); (c) PCL conduit with gold nanoparticles embedded PPEG membrane (AuPPEG membrane) (*n* = 10); and (d) PCL conduits with gold and cobalt-oxide nanoparticle-embedded PPEG membrane (AuCoOPPEG membrane) (*n* = 10). After surgery, the skin was closed and the rats were allowed to recover. Twenty-one days was chosen as sufficient time for matrix formation within the conduit in accordance with a previous study [13].

### 4.6. Harvest of Specimens 

The animals were killed by an overdose of pure pentobarbitalnatrium (Allfatal vet. Pentobarbital (100 mg/mL) (Omnidea AB, Stockholm, Sweden)) after 21 days. The ipsilateral reconstructed sciatic nerve, together with conduit and the formed matrix inside the conduit, as well as the sciatic nerve from contralateral uninjured side and the corresponding dorsal root ganglia (DRG; L4, L5) at the control and experimental sides were removed. Before fixating the tissues in Stefanini solution (4% paraformaldehyde and 1.9% picric acid in 0.1 M phosphate-buffered saline (PBS) pH 7.2), the conduits were carefully removed from the sciatic nerve specimens, leaving only the nerve ends and the matrix that had formed within the conduit; still preserving the original distances. After 24 h of fixation, the samples were washed in 0.01 M PBS, pH 7.2, three times and then placed in a 20% sucrose solution overnight for cryoprotection. The samples were then embedded in OCT Cryo mount (Histolab products AB, Gothenburg, Sweden) and sectioned longitudinally at 6 µm thickness and mounted on super-frost plus glass (Thermo Scientific, Braunschweig, Germany).

### 4.7. Regenerative Matrix

A regenerative matrix was defined as the matrix formed within the conduit that can be observed macroscopically after 21 days of implantation as earlier described [20]. The macroscopic analysis of this regenerative matrix was done in two headings. First, the thickness of the matrix was categorized as: (a) no matrix, (b) thickness < 1 mm (loose) and (c) thickness ≥ 1 mm (dense). Second, the number of full-length regenerative matrix cables that were formed between the proximal and distal ends of the nerve as: (a) no cable, (b) one full length cable and (c) two definable full-length cables.

### 4.8. Immunohistochemistry 

All the immunohistochemistry analyses were conducted on the thickest cable that was present in each rat. The data for second cables is presented separately.

For immunohistochemistry, the sections were washed for 15 min with PBS prior to incubation with primary antibodies. The following primary antibodies were used: monoclonal mouse anti-human neurofilament (1:80; Dako, Glostrup, Denmark), for axonal outgrowth; polyclonal goat anti-HSP-27(1:200, Santa Cruz Biotechnology) for detection of HSP27 in Schwann cells and axons in sciatic nerve; polyclonal rabbit anti-HSP-27 (1:200, Enzo, Farmingdale, NY, USA) for sensory neurons and axons in DRG; monoclonal mouse anti-ATF3 for activated Schwann cells and for sensory neurons in DRG (1:200, Santa Cruz Biotechnology); and monoclonal rabbit anti-cleaved caspase 3 for apoptotic Schwann cells (1:200, Cell signalling Technology) [21]. The antibodies were diluted in 0.25% Triton-X 100 and 0.25% BSA (bovine serum albumin) in PBS. The sections were incubated with antibodies overnight at 4 °C. The next day, the slides were washed with PBS (3 × 5 min) followed by incubation with secondary antibodies; Alexa Fluor 594-goat anti-mouse IgG for the neurofilament immunostaining, Alexa Fluor 488-goat anti-rabbit IgG for the HSP27 on the DRG immunostaining and cleaved caspase 3 on the sciatic nerve, Alexa Fluor 488-goat anti-mouse IgG for the ATF3 immunostaining or Alexa Flour 488-donkey anti-goat for the HSP27 on sciatic nerve immunostaining (all from Invitrogen, Molecular Probes, Eugene, OR, USA), diluted at 1:500 in PBS. For the DRG immunostaining with HSP27, Alexa Fluor 488-goat anti-rabbit IgG was diluted at 1:250 in PBS (Table 5). Sections were incubated with the secondary antibody solution for 1 h in the dark at room temperature. After 1 h, the slides were washed with PBS (3 × 5 min). After washing with PBS, the sections were mounted with Vectashield Mounting Medium with DAPI (4′,6-diamino-2-phenylindole) (Vector Laboratories, Burlingame, CA, USA) and then coverslipped.

### 4.9. Imaging and Analyses 

The sections were photographed using a motorized reflected fluorescence microscope (Olympus BX3) equipped with a digital camera (Olympus DP80) and analyzed with Cell Sens Dimension software (Olympus). In order to decrease the bias in analyses, we have “blind-coded” the slides of each rat. The sciatic nerve sections were photographed at 3 mm distally to the proximal suture site (in the regenerated matrix) and just distal to the distal suture site (distal nerve site) (Figure 3). For the sciatic nerve, the HSP27 stained images were taken under 10× magnification (image size; 1360 × 1024 pixel), and images of ATF3 and cleaved caspase 3 were taken under 20× magnification. DRG sections and the sciatic nerve sections for neurofilament immunostaining were photographed as a whole tissue section using the live process function (Cell Sens Dimension (Olympus)). The length of the positive neurofilament proteins were measured from the proximal suture site to the front of the longest growing axons according to a previous described method [21]. For all immunofluorescence analyses, three random slides were selected from each rat and analyzed and a mean value was calculated for each rat.

#### 4.9.1. HSP27 Immunoreactivity Analysis

After imaging, both the sciatic nerve and the DRG images were imported into ImageJ 1.47 (a public domain image analysis program developed at the U.S. National Institutes of Health, Bethesda, Maryland, USA, http://imagej.nih.gov/, accessed on 23 June 2021). On the sciatic nerve slides, the tissue was marked with a free hand selection tool in order to discard possible artefacts and soft tissues. On the DRG slides, the outermost border of the DRG tissue with sensory neurons was marked, discarding the external nerve tissue. However, nerve tissue that is scattered within the DRG could not be discarded, and was therefore included in the analysis. A labelled region of interest (ROI) (100 × 100 pixels) was selected and placed on the possible darkest area (for the sciatic nerve, darkest nerve tissue furthest away from the transection site; for the DRG, darkest neuronal area). To determine the intensity level of immunohistochemistry, the images were converted to 8-bit grey scale and the tool threshold was used. The intensity threshold was readjusted by adding three times the standard deviation of the background to the mean intensity (mean intensity + 3 × SD). For statistical analysis, the data from L4 and L5 were pooled for each experimental group as well as their control sides. The data for both nerve and DRG were expressed as the percentage of HSP27 immunoreactivity per total selected area of nerve segment or DRG [20].

#### 4.9.2. Analysis of ATF3 and Cleaved Caspase 3 Immunoreactivity:

ATF3 and cleaved caspase 3 stained cells were counted manually in sciatic nerve sections (image size 1360 × 1024 pixel; 20× magnification) both at 3 mm distally from the proximal nerve suture (in the regenerated matrix) and just distal to the distal suture site (distal nerve site) (Figure 3) [21,38,67]. The cells that had a long or oval nuclei and located within the basal lamina parallel to the axons were counted as ATF-labelled Schwann cells as previously described [21,38]. The number of ATF3 and cleaved caspase 3 labelled Schwann cells was expressed as percentage of total number of DAPI-positive cells. At these locations, DAPI positive cells were quantified with ImageJ program. A previously described plugin [68] in ImageJ program was used to quantify DAPI positive cells. The mean values from three slides were taken and expressed as no/mm^2^.

ATF3 analysis on the DRG tissue was performed as follows: ATF-labelled cells on the whole DRG tissue were counted manually. For the analysis of DAPI positive cells in the DRG, the sections were transferred to ImageJ program. For that, similar to HSP27 immunoreactivity analysis, the outermost border of the DRG tissue with sensory neurons was marked with a free-hand selection tool discarding the external nerve tissue. Nerve tissue that was scattered within the DRG was included in the analysis. A previously described plugin [68] in the ImageJ program was used to quantify DAPI positive cells. The number of ATF3 labelled sensory neurons was expressed as percentage of total number of DAPI-positive cells within the selected area. The mean values from three different sections were taken and expressed as no/mm^2^.

### 4.10. Statistical Methods 

All immunoreactivity results are presented as median values (25–75th percentiles) due to a non-normal distribution of data. The nonparametric Kruskal–Wallis (KW) test was used to determine whether there were any significant differences between the four groups with Mann–Whitney U-test as a post hoc test. For the gross analysis (content within the conduit and the number of cables), the chi-square test (Pearson or Fisher’s exact test) was performed. A *p*-value less than 0.05 was accepted as a significant difference. We were interested to find if there is any correlation or association between the axonal outgrowth and cellular activity in the operated sciatic nerve and in the DRGs. For that, we used Spearman’s correlation test on pooled data and linear regression analysis on data that is converted to dummy variables. Statistical analyses were performed using IBM SPSS Statistics software, version 26, New York, NY, USA.

## 5. Conclusions

Gold and gold-cobalt-oxide nanoparticle-embedded PPEG membranes in PCL conduits significantly improve axonal outgrowth compared to hollow conduits across a 10 mm sciatic nerve defect in healthy rats. Activated and apoptotic Schwann cells, i.e., ATF3- and cleaved caspase 3-stained, in the regenerated matrix influenced axonal outgrowth, while HSP27 expression in both the regenerative matrix and in DRG may be more related to neuroprotection rather than directly stimulating axonal outgrowth. Further studies with longer critical nerve defect lengths during longer periods of implantation may clarify whether the modified conduits can be launched for clinical use without any systemic toxic effects.

## Figures and Tables

**Table 1:** Macroscopic analysis of the regenerative matrix is presented as thickness and number of cables formed within conduits that are used to reconstruct a 10 mm long rat sciatic nerve defect.

		PCL Conduits without/with Modifications, i.e., Inserted Membranes				*p-*Values
		Hollow(*n* = 10)	PPEG (*n* = 10)	AuPPEG (*n* = 10)	AuCoOPPEG (*n* = 10)	
Thickness of regenerative matrix (see foot note)	No matrix	4	2	0	0	0.015
	Thickness < 1 mm (loose)	6	4	4	4	
	Thickness ≥ 1 mm (dense)	0	4	6 ^a^	6 ^a^	
Number of cables in regenerative matrix	No cable	4	2	0	0	0.022
	One full length cable	6	4	6	4	
	Two definable full-length cables	0	4	4 ^a^	6 ^a^	

*p*-values as based on Fisher’s exact test and superscript letters indicate the statistical difference between groups with Fisher’s exact test. ^a^ indicates statistical difference against the hollow conduit group. PCL: Poly (ε-caprolactone), PPEG: Poly-propylene polyethylene glycol, AuPPEG:pp Gold nanoparticle embedded PPEG membrane, AuCoOPPEG: Gold and cobalt oxide nanoparticle embedded PPEG.

**Table 2:** Length of axonal outgrowth (NF) and presence of HSP27, ATF3 and cleaved caspase 3 immunoreactivity is presented at 3 mm distal to the proximal site of suture in the regenerated matrix as well as distal to the distal suture site on experimental site and controlateral unoperated sciatic nerve after reconstruction of a 10 mm long rat sciatic nerve defect.

		PCL Conduits without/with Modifications, i.e., Inserted Membranes				*p*-Values
		Hollow (*n* = 10)	PPEG (*n* = 10)	AuPPEG (*n* = 10)	AuCoOPPEG (*n* = 10)	
Length of axonal outgrowth (NF; µm)		4559 (3562–8493)	7169 (4266–9200)	10,113 (5156–12,866)	11,164 (8682–12,366) ^a,b^	0.016
HSP27 immunoreactivity (% of total area) in sciatic nerve at control side		7.2 (5.8–7.6)	6.9 (6.2–7.5)	5.9 (5.0–6.6)	6.2 (5.3–7.7)	0.15
HSP27 immunoreactivity (% of total area) in sciatic nerve at experimental side	At 3 mm	6.9 (4.3–10.5)	11.4 (7.6–15.8)	14.6 (11.6–18.2) ^a^	11.7 (10.1–17.3) ^a^	0.023
	At distal site	11.3 (8.3–13.5)	15.1 (12.2–17.1) ^a^	19.6 (17.5–22.0) ^a,b^	16.1 (13.5–19.6) ^a,b^	0.001
ATF3 immunoreactivity (% of total number of DAPI positive cells)	At 3 mm	3.9 (2.8–5.4)	6.1 (4.9–11.9) ^a^	8.3 (6.2–11.5) ^a^	10.8 (7.2–12.9) ^a^	0.022
	At distal site	5.6 (3.0–9.7)	7.1 (5.3–11)	13.4 (7.5–21.8) ^a^	16.6 (13–23.4) ^a,b^	0.002
Cleaved caspase 3 immunoreactivity (% of total number of DAPI positive cells)	At 3 mm	12.4 (7.5–15.0)	10.2 (8.7–15.1)	8.7 (5.5–10.9)	6 (4.3–7.1) ^a,b,c^	0.004
	At distal site	30.8 (24.4–34.2)	27.2 (23.9–30.7)	25.7 (23.8–29)	24.7 (19.7–28.5)	0.23
DAPI positive cells (no/mm^2^)	At 3 mm	4094 (3513–5753)	3486 (3005–4252)	3472 (2784–4396)	3842 (3020–4413)	0.38
	At distal site	3505 (2849–3776)	3515 (3022–3875)	3692 (2775–4286)	4073 (3767–4381) ^a,b^	0.03

The axonal outgrowth is presented by the length of the neurofilament (NF) staining (µm). HSP27 immunoreactivity is presented as the percentage of the total area (%). The number of ATF3 and cleaved caspase 3-labelledSchwann cells is expressed in percentage of the total number of DAPI positive cells. Data are presented as median (25th–75th percentiles). *p*-values are based on Kruskal–Wallis test with Mann–Whitney U-test as a post hoc test (indicated as superscript letters). ^a^ indicates statistical difference against hollow conduit group; ^b^ indicates statistical difference against PCL with PPEG membrane group; ^c^ indicates statistical difference against PCL with AuPPEG membrane group.

**Table 3:** Immunohistochemical analysis of second cable in the regenerative matrix present in different conduits with and without membranes used to reconstruct a 10 mm nerve defect is presented.

PCL Conduits without/with Modifications, i.e., Inserted Membranes	Length of Axonal Outgrowth (NF; µm)	HSP27 Immunoreactivity (% of Total Area) at 3 mm Proximal Site	ATF3 Immunoreactivity (% of Total Number of DAPI Positive Cells) at 3 mm Proximal Site	Cleaved Caspase 3 Immunoreactivity (% of Total Number of DAPI Positive Cells) at 3 mm Proximal Site
Hollow (*n* = 0)	-	^-^	-	-
PPEG (*n* = 4)	2556 (1351–4161)	13.1 (8.1–19.0)	3.4 (2.1–9.1)	12.6 (5.1–15.2)
AuPPEG (*n* = 4)	2897 (836–4267)	19.3 (17.0–21.9)	4.5 (2.5–7.4)	6.9 (3.2–15.3)
AuCoOPPEG (*n* = 6)	5279 (368–8930)	14.3 (12.3–20.7)	5.8 (2.5–9.6)	5.7 (2.4–8.0)

**Table 4:** HSP27 immunoreactivity and ATF3-labelled sensory neurons in L4 and L5 DRG at experimental and control sides after reconstruction of a 10 mm nerve defect in the rat sciatic nerve.

	PCL Conduits without/with Modifications, i.e., Inserted Membranes				*p* Values
	Hollow (*n* = 10)	PPEG (*n* = 10)	AuPPEG (*n* = 10)	AuCoOPPEG (*n* = 10)	
HSP27 immunoreactivity (control L4 and L5 DRG; %)	3.1 (2.8–3.6)	2.4 (2.1–3.3)	3.0 (2.5–3.6)	2.6 (2.0–3.0)	0.18
HSP27 immunoreactivity (experimental L4 and L5 DRG; %)	9.2 (7.0–11.1)	12.7 (11.2–14.1) ^a^	13.6 (10.9–15.1) ^a^	12.0 (10.7–13.8) ^a^	0.005
HSP27 ratio (Exp/Control)	3.0 (2.0–3.8)	4.35 (3.7–6.7) ^a^	4.3 (4.2–4.9) ^a^	4.5 (3.6–6.7) ^a^	0.015
ATF3-labelled sensory neurons (experimental L4 and L5 DRG; %)	0.8 (0.23–1.6)	1.4 (0.8–2.2)	2.3 (1.3–3.6) ^a^	4.8 (2.9–10.4) ^a,b^	0.002

Data are presented as median (25th–75th percentiles). *p*-values are based on Kruskal–Wallis test with Mann–Whitney U-test as a post hoc test (indicated as superscript letters). ^a^ indicates statistical difference against hollow conduit group; ^b^ indicates statistical difference against PCL with PPEG membrane group.

**Table 5:** The used primary and secondary antibodies for immunohistochemical analysis are listed for sciatic nerve and DRG.

		Primary Antibody	Secondary Antibody
Sciatic nerve	Neurofilament	Monoclonal mouse anti-human neurofilament (1:80; Dako, Glostrup, Denmark)	Alexa Fluor 594-goat anti-mouse IgG (1:500, Invitrogen, Molecular Probes, Eugene, OR, USA)
	Heat Shock Protein 27 (HSP27)	Polyclonal goat anti-HSP-27(1:200, Santa Cruz Biotechnology, Dallas, TX, USA)	Alexa Flour 488-donkey anti-goat IgG (1:500, Invitrogen, Molecular Probes, Eugene, OR, USA)
	Activating transcription factor 3 (ATF3)	Monoclonal mouse anti-ATF3 (1:200, Santa Cruz Biotechnology, Dallas, TX, USA)	Alexa Fluor 488-goat anti-mouse IgG (1:500, Invitrogen, Molecular Probes, Eugene, OR, USA)
	Cleaved caspase 3	Monoclonal rabbit anti-cleaved caspase 3 (1:200, Cell signalling Technology, Denvers, MA, USA).	Alexa Fluor 488-goat anti-rabbit IgG (1:500, Invitrogen, Molecular Probes, Eugene, OR, USA)
Dorsal root ganglia (DRG)	Heat Shock Protein 27 (HSP27)	Polyclonal rabbit anti-HSP-27 (1:200, Enzo, Farmingdale, NY, USA)	Alexa Fluor 488-goat anti-rabbit IgG (1:250, Invitrogen, Molecular Probes, Eugene, OR, USA)
	Activating transcription factor 3 (ATF3)	Monoclonal mouse anti-ATF3 (1:200, Santa Cruz Biotechnology, Dallas, TX, USA)	Alexa Fluor 488-goat anti-mouse IgG (1:500, Invitrogen, Molecular Probes, Eugene, OR, USA)

## Data Availability

The datasets generated and analyzed during the current study are not publicly available, but data can be available for researchers after a special review that includes approval of the research project.

## Supplementary Materials

The following are available online at https://www.mdpi.com/article/10.3390/ijms22137146/s1. Supplementary data 1, EDS analysis of the membranes; Supplementary data 2, SEM and EDS analysis of the rat liver and spleen; Supplementary data 3, Pilot study, S3-Figure 1, S3-Table 1.
