# Supplementary material for: Gold and Cobalt Oxide Nanoparticles Modified Poly-Propylene Poly-Ethylene Glycol Membranes in Poly (ε-Caprolactone) Conduits Enhance Nerve Regeneration in the Sciatic Nerve of Healthy Rats
Source: Int J Mol Sci. 2021 Jul 1;22(13):7146. doi: 10.3390/ijms22137146 (PMC8268459; doi:10.3390/ijms22137146)
Supplement: Supplementary file 1 [file ijms-22-07146-s001.zip › ijms-1252180-supplementary-Revised.pdf]

Pilot study with different types of nanoparticles and membranes

To see the effect of different membrane structures on nerve regeneration, a pilot study was conducted on 10 mm and 15 mm sciatic nerve defect model in rats with different types of nano-structured membranes in PCL conduits. We used amphiphilic copolymer as membranes: polypropylene grafted polyethylene glycol (Mn 2000 g/mol) and poly (N-isopropyl acryl amide) grafted poly (3-hydroxy alkanoate) and gold, silver and a mixture of gold and cobalt-oxide nanoparticles embedded into them.

1. Results

In this pilot study, we detected longer axonal outgrowth with a complete regenerative matrix cable in a 15 mm nerve gap model and two complete regenerative matrix cables in 10 mm nerve gap model in both gold and gold-cobalt nanoparticle-embedded polypropylene polyethylene glycol membrane groups compared to other types of membranes (Table 1—Supplementary Data 3). Statistical analysis could not be performed due to low quantity of data. This indicated a possible superior local neuroregenerative effect of both gold and gold-cobalt nanoparticles. With this information, we decided to further analyze the effect of these nanoparticles on nerve regeneration in modified and unmodified conduits in the present main study.

Table 1. Supplementary data 3: Data for a pilot study using poly-ε caprolactone nerve conduits with inserted membranes as modifications to reconstruct 10- or 15-mm long sciatic nerve defects in rats (only one rat is operated on for each conduit).

Types of modified membranes in PCL conduits	Length of PCL conduit (mm)	Nerve defect length (mm)	Number of regenerated nerve cables	Length of axonal outgrowth (µm)	Thickness of the regenerative matrix (midway, µm)
Silver nanoparticle-embedded poly-propylene polyethylene glycol (AgPPEG)	14	10	2	4221.0/-	576.1/-
	19	15	1	5257.0	425.9
Gold nanoparticle-embedded poly(N-izopropyl acryl amid-poly(3-hydroxy undecenoate (AuPHU-PNIPAM)	14	10	-	-	
	19	15	-	-	
Gold nanoparticle-embedded poly propylene poly-ethylene glycol (AuPPEG)	14	10	2	16,215.0/9876.3	497.9/355.1
	19	15	1	14,411.0	402.9
Gold-Cobalt-oxide nanoparticle embedded poly-propylene poly-ethylene glycol (AuCoOPPEG)	14	10	2	11,280.0/5647.8	360.1/286.5
	19	15	1	14,483.0	608.5

2. Materials and Method:

All the chemical materials used in production of the conduits were supplied by Sigma-Aldrich Chemical company, St. Louis, Missouri, USA. Tetra hydro furan (THF) was passed through a neutral Al_2O_3 column before use. PCL was supplied in granule form with Mn 80,000 g/mol. Chlorinated polypropylene (PP-Cl) has one chlorine atom in average in three repeating units with MW 147 Da. Polyethylene glycol was supplied in MW 2000 Da (PEG-2000). NaH was supplied as 60 wt% in oil.

2.1. Production of poly (ε-caprolactone) (PCL) conduits with modified membranes

Synthesis and production of PCL conduits with polypropylene-polyethylene glycol (PP-g-PEG) membrane and its gold and gold-cobalt modified derivatives are explained in detail in the main manuscript.

2.1.1. Synthesis of PP-g-PEG amphiphilic copolymer with silver nanoparticles

Ag-PP-g-PEG (AgPPEG) nanocomposite was prepared similar to a previous report [33]. The PP-g-PEG graft copolymer (0.81 g) was dissolved in 40 mL of THF. To this solution was added 0.020 g of the $AgNO_3$ in 0.106 g of H_2O, while continuously stirring for 20 min. Furthermore, 0.016 g of NaBH4 in 0.100 g of distilled water was added to this mixture, during continuous stirring. Over the next 30 min, the solution turned to a reddish color.

2.1.2. Synthesis of PHUPNIPAM amphiphilic copolymer with gold nanoparticles

Unsaturated microbial polyester as a natural and biodegradable polyester was obtained from *Pseudomonas oleovorans* growing equally on the mixture of the substrates 10-undecenoic acid and soybean oily acids (a mixture of oleic, linoleic and linolenic acids) according to the procedure described in the cited literature [34-36]. Bromine was added into the double bonds of the PHU solution in CCl_4 according to the procedure described in the cited literature [36]. N-isopropyl acryl amide was purified by crystallization in hexane before use. Xanthogenate functionalized PHU was obtained by stirring with tetra hydro furan solution of the brominated PHU according to the procedure described in the cited literature [37]. Reversible addition fragmentation termination (RAFT) polymerization of NIPAM was carried out in the presence of xanthogenate functionalized PHU in order to prepare PHU-PNIPAM graft co-polymer according to the procedure described in the cited literature [35]. AuPHU-PNIPAM nanocomposite was for the first time prepared similar to a previous report [33]: The PHU-PNIPAM graft copolymer (0.81 g) was dissolved in 40 mL of THF. To this solution was added 0.020 g of $HAuCl_4$ in 0.106 g of H_2O, while continuously stirring for 20 min. Furthermore, 0.016 g $NaBH_4$ in 0.100 g of distilled water was added to this mixture, during continuous stirring. Over the next 30 min, the solution turned violet. The solvent was evaporated and the polymer film was washed with distilled water. The composite film was dried under vacuum at 40 °C.

2.1.3. Synthesis of PP-g-PEG amphiphilic copolymer with gold and gold-cobalt-oxide nanoparticles

This data is presented in detail in the main manuscript.

2.1.4. Modified nano-composite PCL conduit formation

Polymer membranes were prepared using solvent casting from THF [63]. The THF solution of the polymer (AgPPEG, PPEG, AuPHU-PNIPAM, AuPPEG or AuCoOPPEG, 0.87 g in 20 mL of THF) was filtered into a Petri dish (diameter = 5 cm) and the solvent was allowed to evaporate leaving a thin polymer membrane. The solvent cast membrane was submersed into distilled water (200 mL) for 24 h. The polymer membrane was then dried in air at room temperature for 24 h. The air-dried polymer membrane was dried

under vacuum for 48 h for further drying. From each polymer membrane, two sets of rectangular membranes were cut (2 mm width and 10 mm length; and 2 mm width and 15 mm length) were cut and placed into the chitosan conduits. Finally, nano-conduits were formed as follows: 14 mm and 19 mm long PCL conduits with 10 mm and 15 mm long nanocomposite membranes placed parallel to the long axis of the conduit allowing 2 mm on both the proximal and distal ends of the nerve for insertion and suturing of the ends (Figure 1—Supplementary file 3, Table 1—Supplementary file 3).

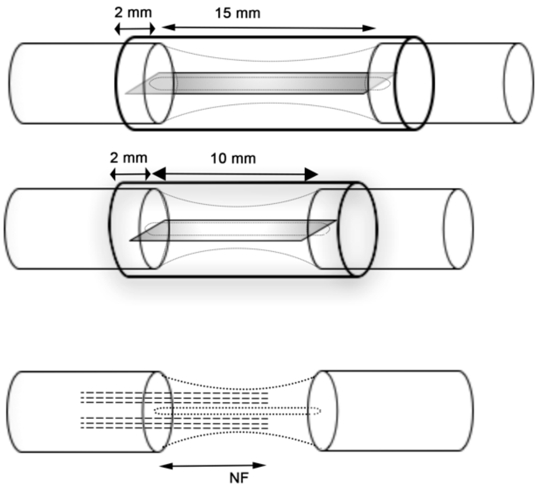

Figure 1. Supplementary data 3. Schematic view of the conduit with inserted polymer membrane used to reconstruct a 10 mm or 15 mm sciatic nerve defect is presented. Nerve regeneration was evaluated at 21 days by immunohistochemistry for axonal outgrowth (length of neurofilaments; NF).

2.2. Animal Surgery

In eight healthy Wistar rats (~200 g), the sciatic nerve was cut and reconstructed with different PCL nerve conduits with inserted membranes and analyzed 21 days after implantation. All animal experiments were approved by the ethical committee in Malmö and Lund region, Sweden (Lund University permit number 06842-19). The details of the animal characteristics, anesthesia, surgery and care are presented in the main manuscript. The sciatic nerve was exposed at the hindlimb level unilaterally and a 5 mm or 10 mm segment of the nerve was removed. A 19-mm or 14-mm-long designed conduit was placed within the nerve defect and 2 mm of the proximal and distal nerve ends was inserted into the conduit (Figure 1—Supplementary Data 3).

2.3. Harvest of the specimens

The methodology of the harvest of the specimens is presented in the main manuscript. The macroscopic analysis of this regenerative matrix is presented as the number of full-length cables that was formed between the proximal and distal ends of the nerve (Table 1—Supplementary Data 3).

2.4. Immunohistochemistry

The formed regenerative matrices within the conduits were analyzed for axonal outgrowth. For immunolabelling, the sections were washed for 15 min with PBS prior to incubation with primary antibodies. For axonal outgrowth, monoclonal mouse anti-human neurofilament (1:80; Dako, Glostrup, Denmark) primary antibody was used [21]. The antibody was diluted in 0.25% Triton-X 100 and 0.25% BSA (bovine serum albumin) in PBS. The sections were incubated with antibodies overnight at 4° C. The next day, the slides were washed with PBS (3 × 5 minutes) followed by incubation with secondary antibody;

Alexa Fluor 594- goat anti-mouse IgG (1:500, Invitrogen, Molecular Probes, Eugene, Oregon, USA). Sections were incubated with the secondary antibody solution for 1 h in the dark at room temperature. After 1 h, the slides were washed with PBS (3 × 5 minutes). After washing with PBS, the sections were mounted with VECTASHIELD Mounting Medium with DAPI (4',6-diamino-2-phenylindole) (Vector Laboratories, Burlingame, CA, USA) and then coverslipped.

2.5. Imaging and analysis

The details for the imaging are described in the main manuscript. The length of the stained neurofilament proteins were measured from the proximal suture site to the front of the longest growing axons according to a previous described method [21]. The thickness of the regenerative matrix is measured at the midway. For all immunofluorescence analyses, three random slides were selected from each rat and analyzed and a mean value was calculated for each rat (Table 1—Supplementary Data 3).
