# Supplementary material for: Gold and Cobalt Oxide Nanoparticles Modified Poly-Propylene Poly-Ethylene Glycol Membranes in Poly (ε-Caprolactone) Conduits Enhance Nerve Regeneration in the Sciatic Nerve of Healthy Rats
Source: Int J Mol Sci. 2021 Jul 1;22(13):7146. doi: 10.3390/ijms22137146 (PMC8268459; doi:10.3390/ijms22137146)
Supplement: Supplementary file 1 [file ijms-22-07146-s001.zip › Supplementary file 1-EDS analysis of the membranes-Au-CoO-PCL- Conduit manuscript.pdf]

Project 1: AuPPEG membrane

22/02/2021 12:44:41

Element	Weight%	Atomic%
C K	76.12	91.70
Cl K	19.56	7.98
Au M	4.32	0.32
Totals	100.00	

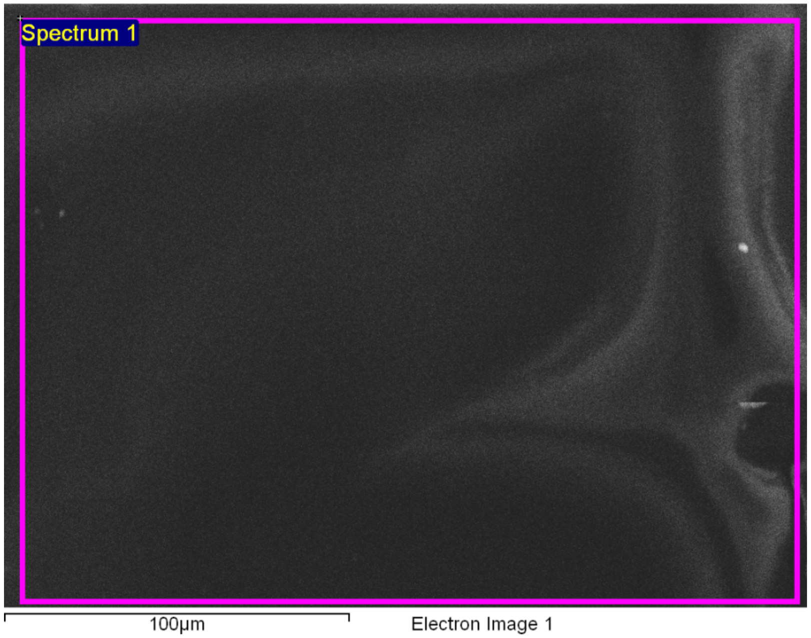

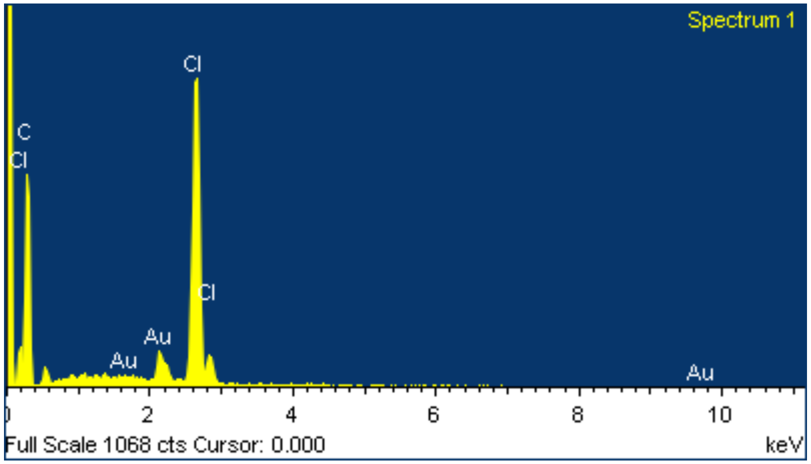

Comment: The analysis of the surface of AuPPEG membrane

Project 2: AuCoOPPEG membrane

22/02/2021 13:03:15

Element	Weight%	Atomic%
C K	71.01	76.91
O K	27.93	22.71
Cl K	1.00	0.37
Co K	0.06	0.01
Totals	100.00	

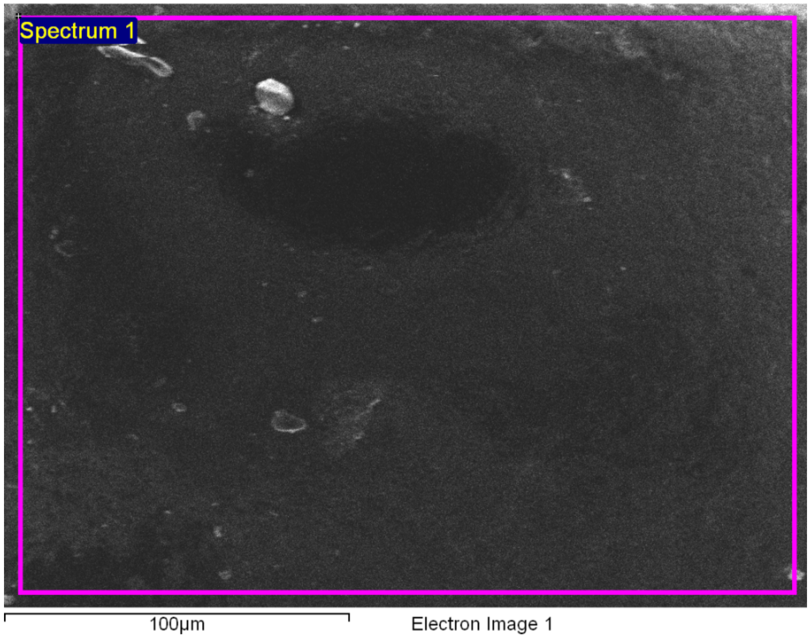

Electron Image 1

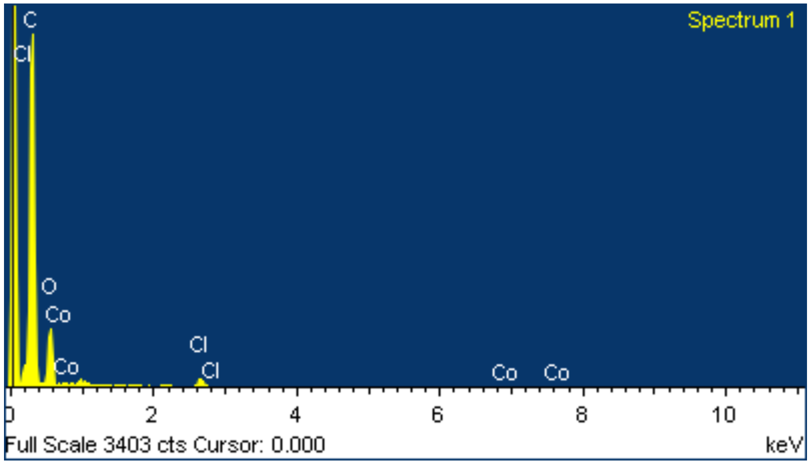

Comment: The analysis of the surface of AuCoOPPEG membrane
