# Supplementary material for: Gold and Cobalt Oxide Nanoparticles Modified Poly-Propylene Poly-Ethylene Glycol Membranes in Poly (ε-Caprolactone) Conduits Enhance Nerve Regeneration in the Sciatic Nerve of Healthy Rats
Source: Int J Mol Sci. 2021 Jul 1;22(13):7146. doi: 10.3390/ijms22137146 (PMC8268459; doi:10.3390/ijms22137146)
Supplement: Supplementary file 1 [file ijms-22-07146-s001.zip › Supplementary file 2-Au-CoO-PCL- Conduit manuscript.pdf]

SEM and EDS analysis of the rat liver and spleen

In order to detect presence of nanoparticles in the rat systemic circulation at the end of 21 days of implantation, we have randomly selected four rats in each of AuPPEG and AuCoOPPEG membrane groups and collected their liver and spleen.

For that: 1 gram liver and spleen tissues were dried under 105^0 C vacume container and were burned under 550^0 C for 30 minutes. A JEOL JSM-6510LV scanning electron microscope (SEM) with Oxford Instruments INCA-energy dispersive X-ray spectroscopy (EDS) for elemental analysis was used to study the morphology of the samples. The specimens were frozen under liquid nitrogen then fractured, mounted, and coated with palladium, gold, and carbon. The SEM was operated at 15 kV, and the electron images were recorded directly from the cathode ray tube on a Polaroid film.

As a result, SEM and EDS analysis did not show any gold or cobalt nanoparticles in liver or spleen tissues of these rats. Here we present SEM and EDS analysis of two rats, from spleen tissue of AuPPEG membrane inserted Rat-24, and from liver tissue of AuCoOPPEG membrane inserted Rat-31.

Project 1-Rat 24

10/25/2018 5:24:01 PM

Element	Weight%	Atomic%
C	17.08	28.04
O	34.44	42.45
Na	2.70	2.31
Al	1.57	1.15
Si	12.50	8.78
P	16.03	10.21
K	8.60	4.34
Ca	1.58	0.78
Fe	5.51	1.95
Totals	100.00	

A JEOL JSM-6510LV scanning electron microscope (SEM) with Oxford Instruments INCA-energy dispersive X-ray spectroscopy (EDS) for elemental analysis was used to study the morphology of the samples. The specimens were frozen under liquid nitrogen then fractured, mounted, and coated with palladium, gold, and carbon. The SEM was operated at 15 kV, and the electron images were recorded directly from the cathode ray tube on a Polaroid film.

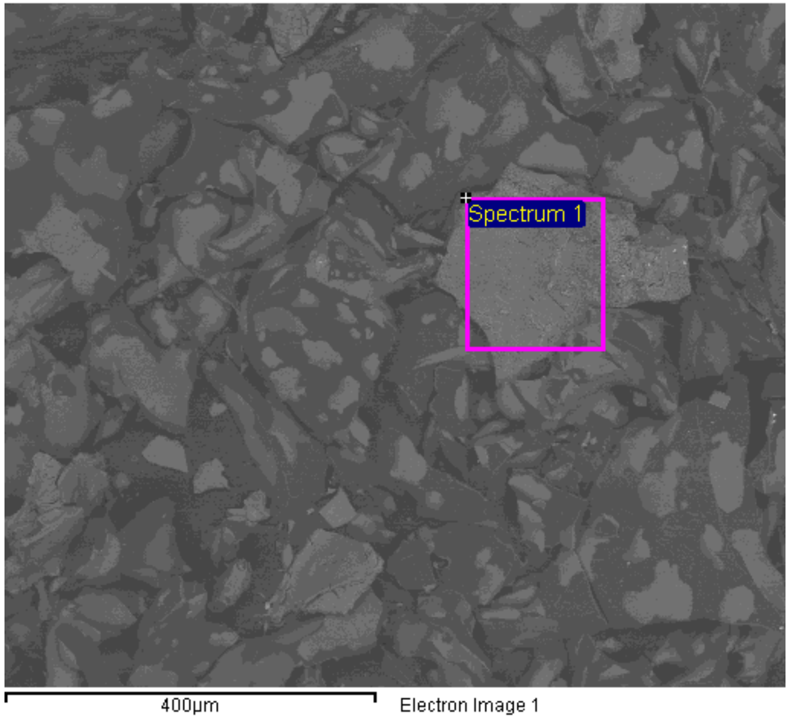

Electron Image 1

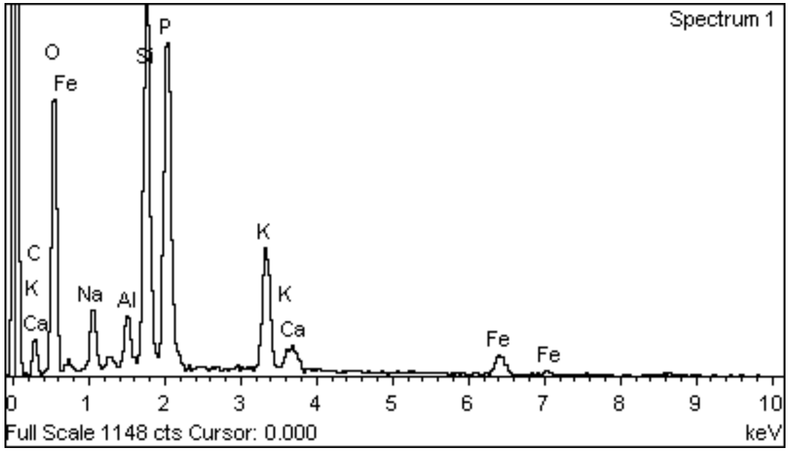

Comment: Spleen-Rat 24

Element	Weight%	Atomic%
C	52.26	64.54
O	27.37	25.38
Na	3.92	2.53
Mg	0.52	0.32
Al	0.49	0.27
Si	2.63	1.39
P	8.57	4.11
K	2.66	1.01
Ca	0.45	0.17
Fe	1.14	0.30
Totals	100.00	

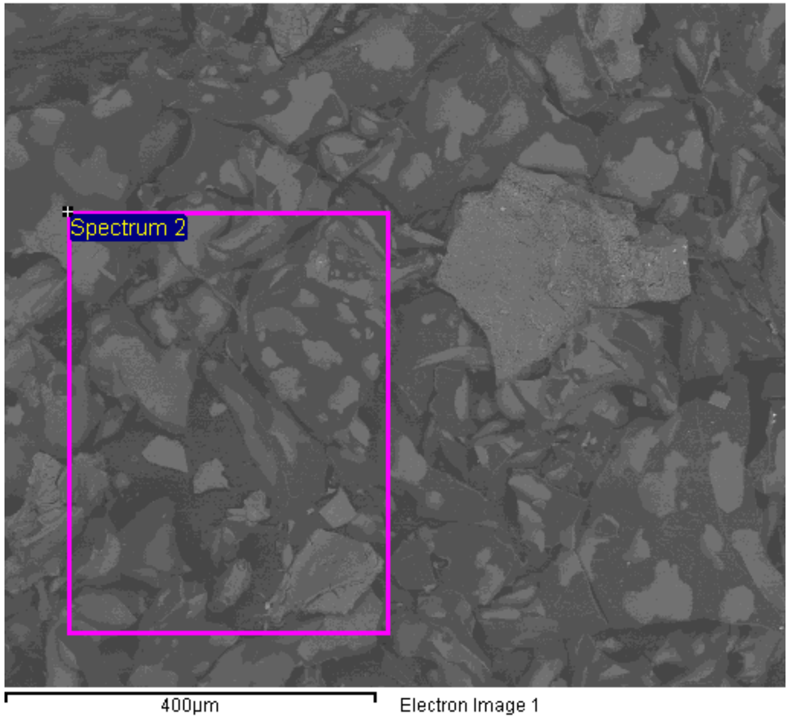

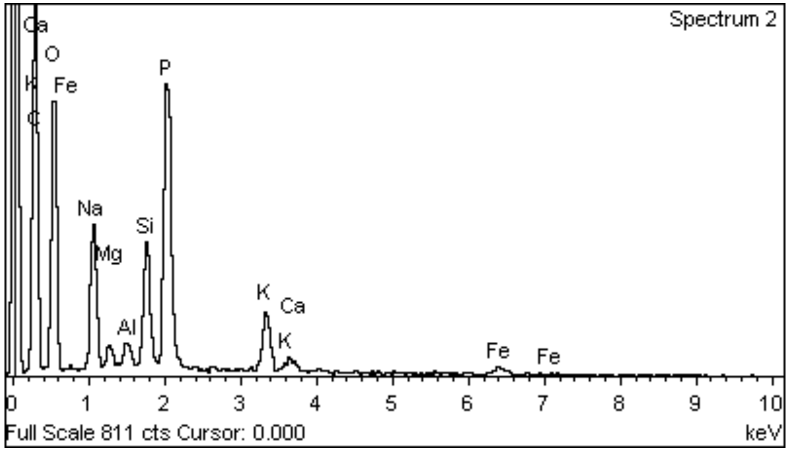

Comment: Spleen-Rat 24

Project 1-Rat 24

Element	Weight%	Atomic%
C	51.01	63.73
O	26.88	25.22
Na	5.59	3.65
Mg	0.64	0.39
P	11.12	5.39
K	2.52	0.97
Ca	0.55	0.21
Fe	1.70	0.46
Totals	100.00	

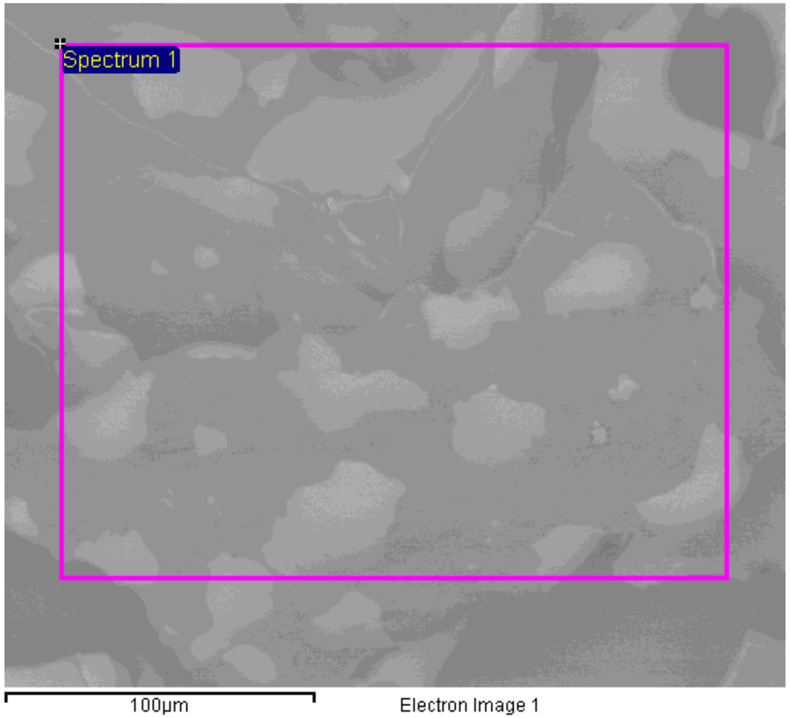

Electron Image 1

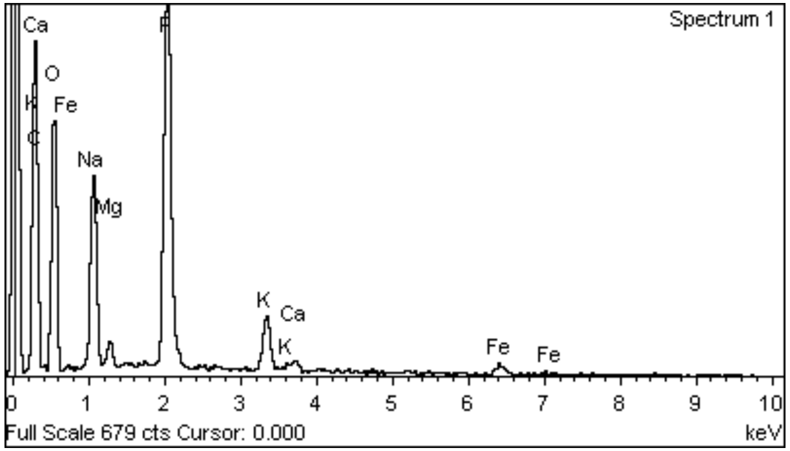

Comment: Spleen-Rat 24

Project 2-Rat 31

Element	Weight%	Atomic%
C	53.21	65.20
O	26.89	24.74
Na	5.32	3.41
Mg	0.51	0.31
P	11.51	5.47
K	1.37	0.52
Ca	0.42	0.15
Fe	0.77	0.20
Totals	100.00	

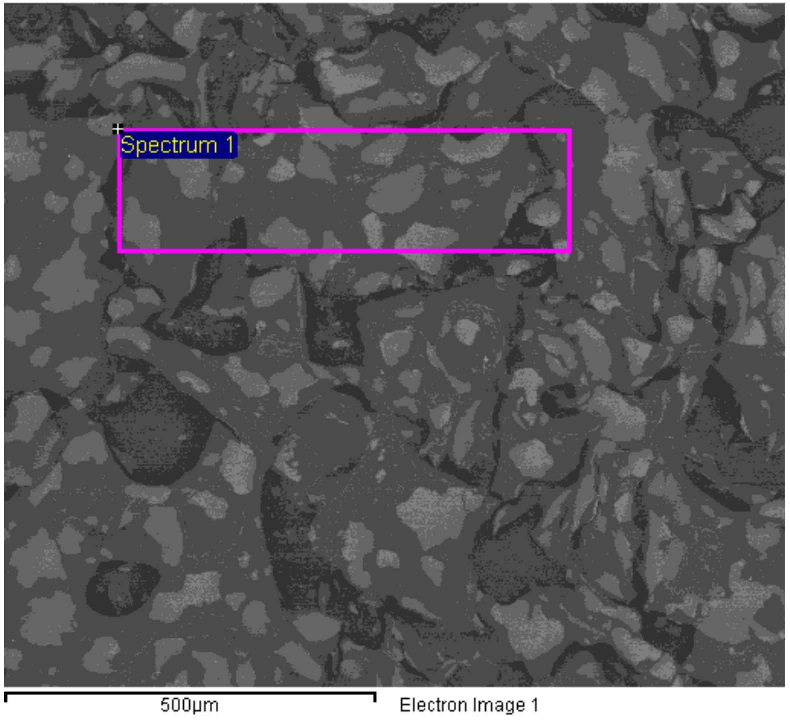

A JEOL JSM-6510LV scanning electron microscope (SEM) with Oxford Instruments INCA-energy dispersive X-ray spectroscopy (EDS) for elemental analysis was used to study the morphology of the samples. The specimens were frozen under liquid nitrogen then fractured, mounted, and coated with palladium, gold, and carbon. The SEM was operated at 15 kV, and the electron images were recorded directly from the cathode ray tube on a Polaroid film.

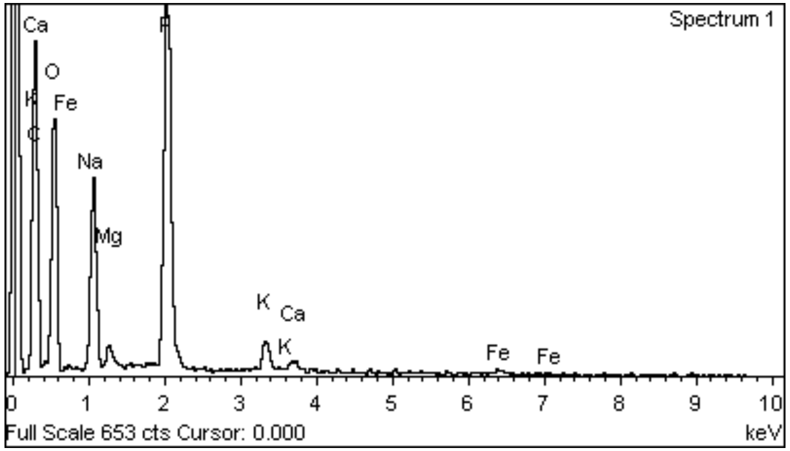

Comment: Liver-Rat 31

Project 2-Rat 31

10/25/2018 5:44:34 PM

Element	Weight%	Atomic%
C	38.98	51.33
O	34.25	33.85
Na	8.00	5.51
Mg	0.79	0.51
P	15.46	7.89
K	1.53	0.62
Fe	0.99	0.28
Totals	100.00	

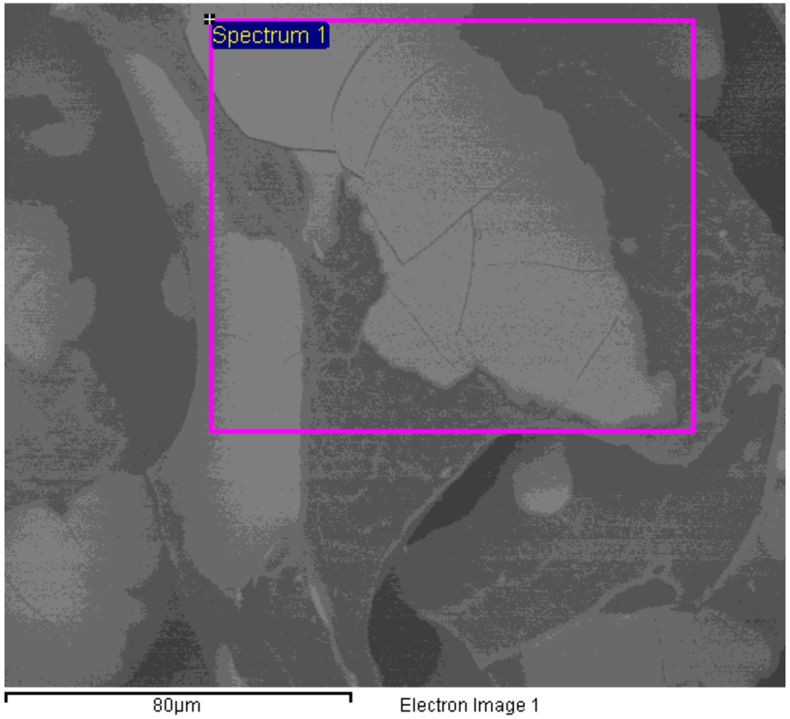

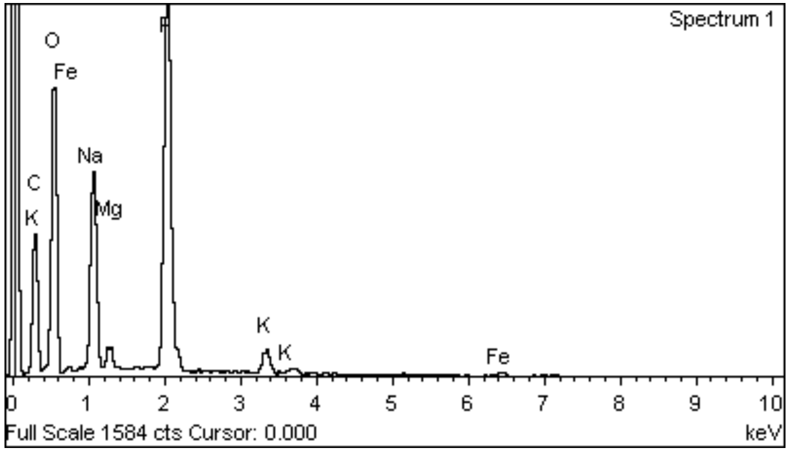

Comment: Liver-Rat 31
